# Current Perspectives in Graphene Oxide-Based Electrochemical Biosensors for Cancer Diagnostics

**DOI:** 10.3390/bios12080607

**Published:** 2022-08-06

**Authors:** Dilsat Ozkan-Ariksoysal

**Affiliations:** Department of Analytical Chemistry, Faculty of Pharmacy, Ege University, Izmir 35100, Turkey; dilsat.ariksoysal@ege.edu.tr; Tel.: +90-232-311-13-53; Fax: +90-232-388-52-58

**Keywords:** electrochemical biosensors, graphene oxide, cancer diagnosis

## Abstract

Since the first commercial biosensor device for blood glucose measurement was introduced in the 1970s, many “biosensor types” have been developed, and this research area remains popular worldwide. In parallel with some global biosensor research reports published in the last decade, including a great deal of literature and industry statistics, it is predicted that biosensor design technologies, including handheld or wearable devices, will be preferred and highly valuable in many areas in the near future. Biosensors using nanoparticles still maintain their very important place in science and technology and are the subject of innovative research projects. Among the nanomaterials, carbon-based ones are considered to be one of the most valuable nanoparticles, especially in the field of electrochemical biosensors. In this context, graphene oxide, which has been used in recent years to increase the electrochemical analysis performance in biosensor designs, has been the subject of this review. In fact, graphene is already foreseen not only for biosensors but also as the nanomaterial of the future in many fields and is therefore drawing research attention. In this review, recent and prominent developments in biosensor technologies using graphene oxide (GO)-based nanomaterials in the field of cancer diagnosis are briefly summarized.

## 1. Introduction

Graphene, called the material of the future, consists of a single layer of carbon atoms arranged in a regular hexagonal lattice appearance [1], is produced from graphite and has led to a wide variety of scientific and technological developments due to its many excellent properties such as conductivity, biocompatibility, unprecedented quality of simultaneous hardness and lightness, etc. Although graphite as a material has been in our lives for centuries, graphene has only recently been recognized as a substance that will change the entire history of science. The history of graphene begins with Geim and Novoselov [2] who won the Nobel Prize in 2010 for their work on this subject. The authors defined graphene as two-dimensional atomic crystals with a carbonaceous structure with promising unique electronic properties [3]. Three of the most well-known and most commonly researched forms of graphene are graphene sheets, graphene oxide (GO), and reduced graphene oxide (rGO) [4].

Since the award of the above-mentioned Nobel Prize, many researchers have been conducting research on graphene in a wide variety of disciplines, including medicine, physics, and chemistry. As of 2022, nearly 296,500 articles based on graphene have been produced and many companies have also started to work in this field. A great deal of effort has been made by researchers on the synthesis of graphene to date, and when these studies are examined in general, it is seen that mechanical exfoliation and chemical vapor deposition (CVD) methods still maintain their popularity. High quality graphene sheets can be produced by both methods [5,6,7]. Since graphene/graphene oxide synthesis is not within the scope of this review and information will be given about graphene oxide-containing biosensors in cancer diagnosis, valuable resources on the subject can be examined from the literature [8,9].

Biosensor research and development, which is a multidisciplinary research area, has been influenced by developments in nanotechnology, especially the use of graphene materials in diagnosis and treatment. Graphene nanomaterials have attracted great interest for many biosensor designs by evaluating both the above-mentioned physical properties, large surface/volume ratio, high biomolecule immobilisation capacities, and wide electrochemical potentials [10]. Graphene, which has a single-layer structure and carbon atoms on its surface, has the feature of interacting directly with biological materials. Another important feature of this material is that some drugs, such as aromatic chemotherapy drugs, can be loaded on its surface. This charge can be realized owing to its delocalized π electrons [11]. In addition, in line with the studies carried out to date, graphene and its derivatives are considered to have the largest surface area compared to most other nanomaterials due to their 2D planar nature (approximately 2600 m^2^ g^−1^) [12]. 

Since there is a high rate of binding to the interfacial area of graphene for many biomolecules such as enzymes, antibodies, cells, proteins, and DNA [13,14], to date, there are numerous applications of graphene and its derivatives, including GO, rGO, Gr quantum dots, Gr nanoplates, and Gr nanoribbon, in many fields, from energy devices to water purification, DNA sequencing to biosensors. Within the scope of this review, prominent graphene oxide-containing biosensor studies in medicine, especially for cancer diagnosis, are included. 

### 1.1. Graphene Oxide (GO)

As is known, graphene oxide can structurally carry oxygen-containing functional groups (i.e., hydroxyl, epoxy, etc.). Apart from these groups, there may be addition of carboxyl functional group at the edges of the graphene layer [15]. On evaluation of the graphene oxide structure considering these aspects, it has been determined to consist of sp^2^ and sp^3^ hybridized carbon atoms and that there is also a random arrangement of oxygen atoms on its surface due to its amorphous structure [16]. Oxygen functional groups carried by GO provide suitable sites to bind various structures, such as polymers, nanomaterials, etc., to the sensing surface, either covalently or by adsorption. However, the efficiency of adsorption especially depends on the nanoparticle and biomolecule concentration [17,18]. Another advantage of GO is that it often has better solubility than graphene under suitable experimental conditions [19,20,21,22]. Of course, the main role of the graphene oxide usage in biosensor schemes is for signal amplification and enrichment of the sensing surface area, just as with many nanomaterials.

Another groundbreaking feature is the demonstration that the GO-COOH material has intrinsic peroxidase-like activity [23]. Following this invention, it has been determined in various studies conducted for glucose, H_2_O_2_, or SNP determination that fullerenes, carbon nanotubes, mesoporous carbon, and even carbon nanodots have similar catalytic activity [24]. As is known, peroxidases are used for H_2_O_2_ scavenge, and if we take HRP for example, this enzyme catalyzes the conversion from H_2_O_2_ to H_2_O by transferring two electrons from an electron-donating substrate [25]. Similarly, GO-COOH can also catalyze the reduction of H_2_O_2_, and the reaction usually takes place with TMB (3,3,5,5-tetramethylbenzidine), a typical substrate used in these studies [23]. 

### 1.2. Reduced Graphene Oxide (rGO)

In the synthesis of GO using the Hummers method mentioned above, some damage occurs in the sp^2^ plane of graphene due to the oxygen-containing functional groups on the surface. This situation causes decreased conductivity of the GO. Low conductivity can be a problem especially in electrochemical biosensor designs. To solve this problem, reduced graphene oxide (rGO) can be modified directly onto the transducer surface, or GO reduction can be done thermally, chemically, or electrochemically prior to use. Thus, conductivity, which was reduced during GO synthesis, is largely regained after reduction from GO to rGO [10]. The rGO obtained as a result of reduction also has a partially restored sp^2^ lattice, although it also contains oxygen-carrying groups to some extent [15,26].

The diversity of biosensing strategies has developed in response to the evaluation of all these general properties and advantages of graphene oxide nanomaterials, especially in the last decade, attracting the attention of both academia and industry and encouraging researchers to prepare biosensor platforms that can be converted into devices. 

### 1.3. Graphene Oxide Based Biosensors: Background

To the present day, graphene oxide has been involved in biosensor designs in many different areas for the detection of, e.g., DNA [27,28,29], proteins [30,31], drugs [32,33,34], glucose [35], bacteria [36,37] viruses, [38], metal ions [39], and various types of biomarkers, including protein, dopamine, microRNA etc. [40,41]. 

Before moving on to the examples of biomolecular sensing within the scope of this review, it would be appropriate to give information about the affinity of biomolecules to the graphene oxide surface in this section.

#### 1.3.1. Graphene Oxide–Biomolecule Interactions

In general, biomolecule immobilizations can be made on the surface of graphene and its derivative nanomaterials through both covalent and non-covalent interactions [42,43]. However, in this section, evaluations will be made on graphene oxide.

As mentioned briefly in Section 1.1, it has been determined that GO nanomaterial is soluble in water and in suitable solvents, thanks to some functional groups (carboxyl, hydroxyl, carbonyl, epoxy) on the graphene oxide surface. These groups also show high affinity to water molecules [44]. The interaction of a single-stranded DNA probe with GO was found to be based on π–π stacking. The findings obtained by fluorescence, Nuclear Magnetic Resonance (NMR), etc., also show that DNA is rapidly adsorbed on the GO surface [45]. However, thanks to the oxygen-carrying groups on its surface, it is also possible to bound the DNA to the GO surface by forming a self-assembled monolayer structure through covalent interactions via carbodiimide chemistry. Similarly, “antibodies”, another biomolecule that can take place in the biosensor sensing unit, are attached to the graphene oxide surface by EDC/NHS chemistry technique, while if an enzyme molecule is in question, it is usually bound by physisorption [46]. Apart from these, many important functionalizations, such as quantum dots, various metal nanomaterials, and metal doping processes in covalent and non-covalent ways, are also used in sensing-surface preparation in biosensor designs containing graphene oxide. Comprehensive information on all these important functionalizations is presented in the review by Georgakilas et al. [47]. 

On the other hand, in cases where graphene oxide is not used as an electrode modifier, biomolecules are immobilized on carbon or metal-based electrode surfaces. The most basic techniques for the immobilization of the biorecognition element in biosensor studies in this context; physical/ electrostatic adsorption, covalent bonding, such as surface activation with EDC/NHS, self-assembled monolayers (SAM) with thiol-labeled short nucleic acids-based interactions, and avidin–biotin interactions are preferred [48,49]. For biomaterial immobilization to the surface in the physical adsorption method, the probe biomolecule (i.e., DNA) solution is contacted with the electrode surface for an appropriate time (the solution is applied to the electrode). The probe is connected to the surface weakly and horizontally. It is the simplest method in terms of fixing the capture DNA and it is very easy to be applied to automation. The method eliminates the use of expensive chemicals and steps such as electrochemical potential application. Therefore, it is cheap and shortens the determination period [50]. However, this technique is not suitable for the clinical diagnosis applications because of its low stability due to the desorption of biomolecules (i.e., aptamers) from the sensor surface [51].

In electrostatic biomolecule immobilization to the surface, (+) positive potential is applied to the electrode surface from the electrochemical transducer system. Thus, DNA with a (−) charged phosphate backbone attaches parallel to the surface by means of electrical attraction forces [52].

In the development of electrochemical DNA biosensors and DNA microarray devices, fixation of the relevant biomolecule to the transducer system surface using adsorption methods is simple and economical. Since these methods do not require complex surface chemistry applications, they are highly compatible with biosensor systems. However, these methods have some limitations such as surface stability and reproducibility.

When a strong binding to the sensor surface is desired, the capture probe biomolecule (i.e., DNA) must be fixed on the surface by covalent attachment (chemical bonding) at one or more points. Preferably, it is more convenient to attach the related DNA from a single point and vertically in terms of structural freedom. Another thing to consider is the presence of a 3–6 carbon-long alkyl group (linker) between the target and the surface of the probe helix. The sensitivity of the biosensor can be increased by this intermediate layer. Amino, thiol, and biotin groups can be added to the end of the alkyl chain according to the design feature [53]. In this way, a vertical, regular, free, and ordered probe DNA layer (SAM) is obtained on the electrode surface. In addition, by adding chemicals, which can also be called diluents, to the spaces between these probe lines, they can be modified more sparsely and perpendicularly to the electrode surface. (e.g., mercaptohexanol, ethanolamine, bovine serum albumin, etc.). Thus, DNA hybridization efficiency is increased.

Carbodiimide chemistries are frequently used for covalent attachment of DNA [54,55,56,57,58,59]. Using mercaptoalkyls (3-mercaptopropionic acid, mercaptohexanol, or l-cysteine/cysteamine, etc.) for gold (Au) electrodes (solid gold electrodes, disposable screen-printed gold electrodes), and gold nanoparticle coated surfaces, the thiol (–SH) in these materials) groups are fixed on the electrode surface [48,57,58,60,61]. This method exploits the strong affinity of thiol groups for gold metal. Then, by using covalent agents such as N-Hydroxy succinimide (NHS) and ethyl carbodiimide (EDC), a basis is created that allows the nucleic acid probe containing the amino group to be firmly attached to the surface. Another method is that the probes labeled with the thiol group from the tip can be fixed to the electrode in a single operation by directly interacting with the surface [60,62]. These two methods are biomolecule binding pathways specific to the gold electrode. Covalent probe attachment to the carbon (graphite) electrode surface is accomplished using carbodiimide (EDC) and succinimide (NHS). After the surface is coated with these materials, the probe marked with the amino group is fixed vertically on the surface [54,55,56]. On various electrode surfaces coated with streptavidin or on surfaces such as magnetic beads, biotin-labeled probes are specifically and vertically attached due to the strong affinity of streptavidin and biotin [63,64]. However, the use of binder- and functional-group-added probes for electrochemical biosensors brings an additional cost and increases the analysis time. Despite the advantages and disadvantages mentioned above, covalent bonding and the use of related agents is the most preferred method in cancer diagnosis with electrochemical biosensor technology. 

#### 1.3.2. Electrochemical Techniques for Cancer Markers Detection

Electrochemical impedance spectroscopy (EIS) is a technique that allows the analysis of both the resistive and capacitive properties of physical and/or biomolecular modifications of electrode surfaces, including nanomaterials, nucleic acids, proteins structures etc. In this concept, EIS is one of the basic electrochemical methods used to determine the fundamental redox events at the electrode–electrolyte interface. In fact, it is the most widely used method in studies of this type of research. However, evaluations are usually made on the basis of complementary results obtained with both the EIS and CV method. For example, in a study carried out by Gong et al. to investigate the properties changing on the surface with graphene oxide modification, Nyquist curves obtained from the solution interface with the glacial carbon electrode using the conventional redox probe ferri/ferrocyanide were evaluated [65]. The electrode/electrolyte solution resistance (Rs) and Warburg impedance (Zw) in the Randles equivalent circuit generally represent the properties of the solution and the diffusion of the redox probe used on the electrode surface and are not affected by the reaction occurring on the electrode surface. The other two elements of the equivalent circuit, the double layer capacitance (Cdl) and electron transfer resistance (Ret), depend on the dielectric and insulating properties at the electrode/electrolyte interface. Electron transfer process over Ret, that is, [Fe(CN)_6_]^3−/4−^, which is the parameter especially focused on in impedimetric studies, is strongly affected by electrode modification. In line with this basic information, a Nyquist curve with a very small semi-circle was obtained in the presence of a redox probe with the glassy carbon electrode (GCE) electrode used, and then an increase in the diameter of the semi-circle was observed when the experiment was repeated with the GO modified electrode. This has been interpreted as the modification of GO, whose sp^2^ networks are disrupted during its synthesis, and that electron transfer to the electrode interface is prevented, and this layer acts as an insulating layer. Then, a single-stranded DNA was covalently immobilized on the GO modified electrode surface and it was observed that the Ret value increased further. It is stated that this situation occurs due to the electrostatic repulsion of the negatively charged phosphate backbone in DNA against ferrocyanide. Then, a single-stranded DNA was covalently immobilized on the GO modified electrode surface and it was observed that the Ret value increased further. This situation is evaluated as both the DNA binding to the surface and the negatively charged phosphate backbone of DNA exerting electrostatic repulsion against ferrocyanide. In the continuation of the experiment, when the electrode surface was subjected to electrochemical reduction (when scanning from 0.0 to −1.7 V) after DNA modification, it was determined that the Ret value decreased dramatically, that is, the conductivity increased clearly. It is an indication that the GO on the surface decreases as a result of reduction and this situation occurs with the formation of rGO. To summarize, the use of rGO in biosensor platforms is due to its contribution to the acceleration of electron transfer. It has been proven by many studies that it improves EIS responses in DNA biosensor designs. When hybridization occurs, the Ret value increases again because this means that [Fe(CN)_6_]^3−/4−^ cannot penetrate the double helix bundles with more negatively charged phosphate backbones on the surface. Similarly, in CV experiments performed with step-by-step surface modifications and with the same redox probe, the findings obtained from the electrode-electrolyte interface are compatible with the EIS results. 

In fact, many of the GO production methods are known to damage this structure. As a result of this damage, it was determined that some functional groups were formed in the substance or on its surface, and carbonaceous or metallic impurities were formed. However, it is seen in many reports that this situation is often turned into an advantage by electrochemical or chemical means. For example, covalent bonding procedures, [56] which are almost traditional for biosensor studies, can be carried out in the similar chemical reactions at the electrde–electrolyte interfaces on GO modified surfaces, thanks to the functional groups on the GO surface. In addition, in the last decade, graphene-friendly linkage molecules have been used in surface preparation in biosensor designs [66].

CV, DPV, and SWV techniques are also used in both label-free and label-based biosensors to monitor the redox events and mechanisms occurring on the electrode surface. Of these, cyclic voltammetry (CV) is the most widely used voltammetric method to explain basic electrochemical events, such as the thermodynamics of oxidation-reduction reactions and electron-transfer kinetics occurring at the electrode-solution interface, as well as mass transport towards the electrode surface [67,68]. In this technique, the current change is measured while the potential applied to the working electrode is scanned from initial potential (E1) to end potential (E2) and from E2 to E1 (or by scanning the potential positive to negative and negative to positive back and forth between selected limits). The comprehensive information from the CV can be used to obtain qualitative information about the electrochemistry of chemical or biological materials (potentials at which the material is oxidized and reduced, peak heights) as well as nanomaterial modifications to the electrode surface. In many biosensor studies, traditional redox probe [Fe(CN)_6_]^3−/4−^ is used for label-free analyses as given in the following sections. 

Differential pulse voltammetry (DPV) is another basic electrochemical technique and in a cell containing a triple electrode system, measurements are performed at the moment when the faradaic current is highest and capacitive current is lowest. Thus, the signal-to-noise ratio increases and hence sensitivity increases. In the method, the difference in current per applied pulse (∆Ip) is recorded as a function of linearly increasing potential. The resulting differential curve is in the form of a peak and its height is proportional to the concentration [69].

Square wave voltammetry (SWV) is also a pulse voltammetry technique that has the advantage of being extremely fast and at the same time sensitive. In this method, a potential is applied to the working electrode in the form of a symmetrical square wave. In each square wave cycle, the current is measured twice, at the end of the applied forward and reverse pulses. The difference of these currents gives the net current value. The square wave voltammogram is obtained when the net current obtained is plotted as a function of the applied potential. The SWV measurement is extremely fast, it is possible to increase the precision of the analysis by averaging several voltammetric measurements [68].

As a result, the modification and redox events occurring on the electrode surface can be elucidated by evaluating these techniques individually or together.

### 1.4. Graphene-Based Electrochemical Biosensor Strategies for Cancer Cells or Biomarker Detection

Cancer is among the diseases that carry a serious risk of death, and it is becoming more and more common around the world. There are about 200 different forms of cancer detected so far [70]. According to the data in the 2020 reports prepared by citing the World Health Organization databases, the types of cancer with high cases number diagnosed are breast (2.26 million), lung (2.21 million), and prostate (1.41 million cases) cancers. In addition, these studies revealed that approximately 10.0 million (95% UI: 9.7–10.2 million) people worldwide died from cancer diseases in 2020 [71].

**Figure 1:**
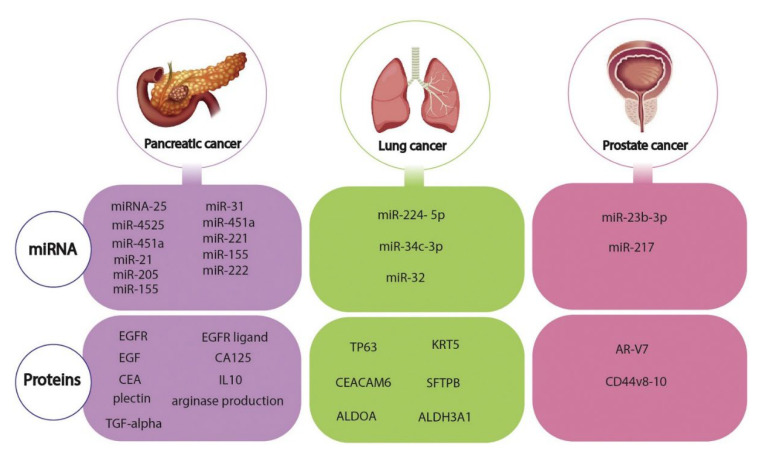
Diagram showing the miRNAs and protein-based pancreatic, lung and prostate cancer biomarkers. Reprinted with permission from Ref [73]. Copyright 2022, Elsevier.

Since early diagnosis in cancer is life-saving, new diagnostic technologies to be developed must provide fast and accurate analysis. In this context, cancer biomarkers are becoming increasingly attractive targets for electrochemical biosensor designs alongside classical detection methods, as they are useful tools for understanding the developmental processes of cancer disease. Thanks to advances in nanotechnology, various cancer biomarkers, i.e., cancer cells, metabolites, miRNA, surface antigens/proteins, etc., biodetection is possible [72]. Some of the critical biomarkers for cancer diagnosis are shown in Figure 1 [73] and also in reference [72].

However, there are some difficulties in developing a biodevice with features that will allow early detection of cancer. For example, the biosensor should be able to perform sensitive and selective analysis with a small sample. If an evaluation is made in terms of the number of cancerous cells, it is expected that a biosensor with clinical validity will accurately detect a tumor in the range of 100–1000 cell counts [74]. Despite all these difficulties, many biosensors containing optical, piezoelectric, and electrochemical transducers continue to be developed as strong alternatives to classical analysis methods for cancer diagnosis. Among the biosensor types [75,76], electrochemical biosensors have competed with optical ones, which are quite advanced in this field, for the analysis of cancer biomarkers, thanks to their qualified features such as ultra-sensitivity, fast response, selectivity, ease of use, low-cost design, and miniaturization [72,73,74,75,76,77]. Electrode types that best incorporate these advantages in their infrastructure are SPE, microelectrodes, or microfluidics electrochemical systems (paper-based electrodes). The most striking electrode types in biosensor designs are those produced with screen printed technology. This technology, which emerged as the printing of conductive powders on a ceramic material surface with the help of an adhesive chemical, is now successfully used in both biomedical and environmental analysis with the production of paper-based and wearable sensor electrodes [78]. The prominent development at this point is the combination of SPEs with simple paper-based microfluidics (µ-PEI), which has many advantages in the design of electrochemical biosensors compared to conventional SPE and other analytical devices. For example, paper-based electrodes are easy to manufacture and use, inexpensive, and suitable for biomaterial/nanomaterial modifications for biosensing applications [79] including cancer diagnosis [80]. 

In this section, prominent examples of graphene-containing electrochemical biosensors developed for cancer diagnosis will be given. It was determined that real samples (for example, human serum, diluted human serum, controlled cancer cells, or samples with microRNA added, etc.) analyses were also performed in all of the studies in the review. Some other interesting designs and the basic contents of them are collected in two different tables at the end of the review (please see Table 1 and Table 2). 

#### 1.4.1. Label/Redox Mediator/Indicator-Based Electrochemical Biosensors

In this part of the review, the section called “label-based biosensors” includes studies where a redox mediator, an electroactive indicator, an enzyme label, a dye, etc. were used in biosensor design and the electrochemical response was obtained thanks to this label. 

**Figure 2:**
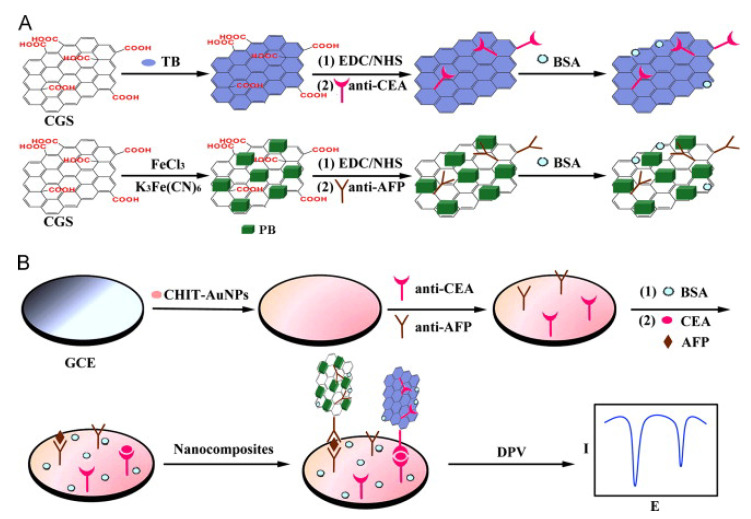
(**A**) The separate preparation of TB, CEA, and AFP type antibody-carrying carboxyl graphene nanosheets (CGS). (**B**) Process steps in the detection strategy of the developed biosensor; Chitosan and gold nanoparticle modification, capture antibody immobilization, blocking with BSA, incubation with specific antigens, interaction with biofunctional CGS nanocomposites, and measurement by DPV method. Reprinted with permission from Ref. [81]. Copyright 2013, Elsevier.

An interesting design containing graphene was performed by Chen et al. [81] for the simultaneous detection of carcinoembryonic antigen (CEA) and alpha-fetoprotein (AFP) based on sandwich immunosensing process, indicators, and differential pulse voltammetry. In this study, carboxyl graphene nanosheets (CGS) were used as a “carrier” for both indicator materials and antibodies. It also provided a large surface area as a modifier of the transducer system, thus allowing a large amount of biomolecules to be attached to the sensor. In the experimental process steps (Figure 2), it is seen that a bioconjugate “signaling probe” structure is prepared by interacting both the indicator and antibodies with CGS. In this process, one of the immunosensor probes was prepared by labeling with anti-CEA following the immobilization of toluidine blue (TB) substance to CGS, and the other by labeling with anti-AFP following the immobilization of Prussian blue (PB). Then, in the sensor fabrication process, chitosan and gold nanoparticle modification and capture antibody immobilization were performed on the glassy carbon electrode surface (GCE), respectively. The analysis was carried out by incubating the modified GCE electrode surface with various concentrations of CEA and AFP antigen mixtures and then interacting with signaling probes containing graphene, antibodies, and electroactive dye molecules. Thanks to the peak currents obtained from TB and PB at different potentials with the DPV technique, simultaneous determination was performed both in CEA or AFP solutions prepared in the laboratory and in real serum samples (linear range 0.5–60 ng/mL).

The fact that there is no need for signal amplification in the detection scheme of the developed immunosensor makes the design interesting and clinically applicable. Because, although low detection limits were obtained only with amplification in many studies, the detection limits were calculated as 0.1 ng/mL for CEA and 0.05 ng/mL for AFP with non-amplification procedures in this study. These data show that the developed biosensor can be an alternative to conventional detection systems in meeting the low detection limit requirement expected in clinical diagnosis. In addition, it has been stated that after surface modification, if the immunosensor is kept under suitable conditions and stored at refrigerator temperature, it can perform the analysis with only 25 percent signal loss even after 30 days.

Another interesting immunosensor platform was developed by Shiddiky et al. [82] for the sensitive and selective analysis of a cancer biomarker (Epithelial cell adhesion molecule, EpCAM) in human serum. The biosensor scheme includes a modified hydrophilic polyacrylic acid brush (PAA) as an antifouling agent on the ITO substrate and a special “bionanoconjugate” containing graphene, quantum dots (QDs), and antibody complex as a signal probe. In this study, it was aimed to prevent non-specific antibody adsorption onto the biosensing surface and to reach very low detection limits by using PAA. For this purpose, EPCAM antibody was attached to the ITO surface by applying PAA immobilization by polymerization and standard carbodiimide chemistry processes, respectively. After the immunoreaction between the EPCAM antibody and antigen, interaction was achieved with a second biotin-labeled EPCAM antibody at the ITO surface. Because this second antibody is biotin-labeled, it can bind to both CdSe QDs and streptavidin-modified two-dimensional graphene oxide (2D-GR) nanolayers. This particular nanomaterial is designed as a signaling probe, which forms the basis of the detection strategy in the developed biosensor. Finally, to monitor the antibody–antigen binding event, these CdSe QDs were dissolved in acidic solution and the electrochemical response of Cd^2+^ ions transferred into solution was determined using a glassy carbon electrode (GCE) and square wave anodic stripping voltammetry (SWASV). It is understood that this methodology, which was discovered by Professor Wang and his team in 2002 and is based on the dissolution of metal ions from QDs with acidic solution, is still a popular and powerful alternative electrochemical biosensor design technique despite many years since its discovery [83,84]. Analyses with the developed immunosensor were performed in both phosphate buffer and human serum media, and the limits of detection (LOD) were estimated as 5 fg/mL and 150 fg/mL, respectively. The reproducibility of the responses obtained with the four immunosensors prepared just before the experiment using 1 ng/mL EpCAM was 6.8% (n = 4).

**Figure 3:**
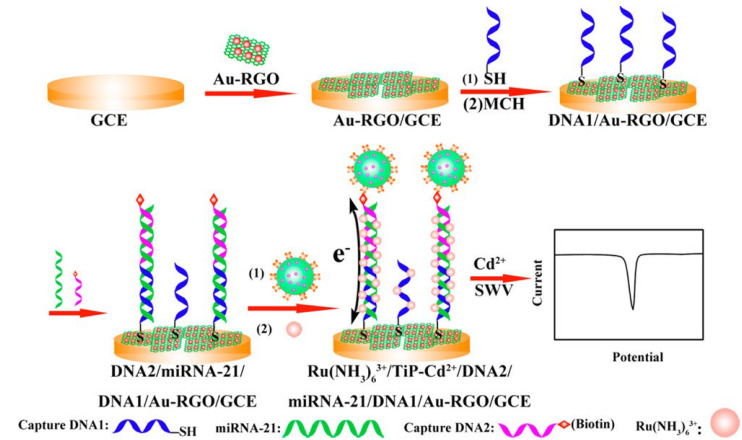
Modification of the GCE electrode surface with nanomaterials and experimental steps related to sandwich type detection. Reprinted with permission from Ref. [85]. Copyright 2015, American Chemical Society.

As it is known, in this classical sensing method, obtaining the electrochemical signal basically depends on the dissolution of the quantum dots in an acid solution. As an alternative technique, to eliminate the associated dissolution process, Zhu and his research group synthesized metal-ion-functionalized titanium phosphate nanospheres (TiP) and used them in the form of TiP−Cd^2+^ conjugates as a label that can be used directly in the analysis [85]. In the study in which the related research group performed microRNA (miRNA-21) analysis at the attomolar level, graphene oxide- and gold-nanoparticle-modified glassy carbon electrode were used as transducer and Ru(NH_3_)_6_^3+^ was used as electron transfer mediator. A sensitive analysis was carried out since a high amount of Cd^2+^ was modified on TiP nanoparticles, which will provide the voltammetric signal of Cd^2+^ without need for acid dissolution, and the electrochemical response increased more than 5 times with the synergistic effect of ruthenium molecule (Figure 3). It is understood from the obtained results that this biosensor system, which includes two capture probes and operates according to the sandwich assay protocol, will provide an ultra-sensitive platform for miRNA analysis in clinical diagnostics.

**Figure 4:**
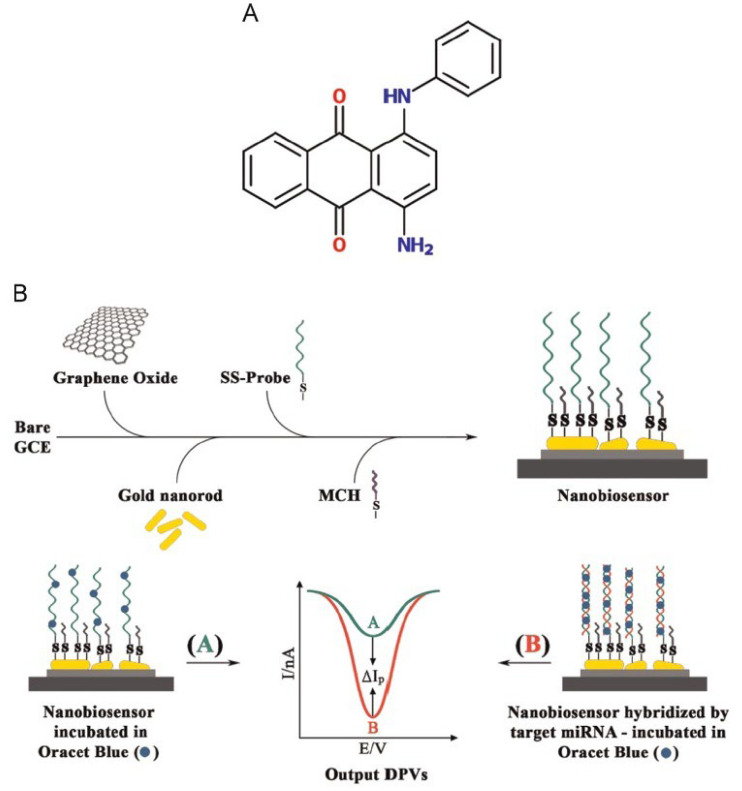
(**A**) Chemical structure of the Oracet Blue indicator. (**B**) Schematic representation of the process steps of the surface modification and working procedure of the electrochemical nanobiosensor developed for miR-155 determination. Reprinted with permission from Ref. [86]. Copyright 2016, Elsevier.

Azimzadeh et al. [86] performed sensitive and selective detection of the breast cancer biomarker miR-155 with a DNA biosensor prepared by modifying the graphene oxide layer with gold nanorods (GNRs). Incubation was provided for hybridization between target miRNA and probe DNA on the prepared nanobiosensor surface (Figure 4). In the determination, the difference in the signals obtained before and after hybridization was evaluated by using the electrochemical measurement technique DPV and an electroactive indicator molecule “Oracet Blue (OB)”. The biosensing layer prepared on the glassy carbon electrode surface consists of graphene oxide, gold nanorod, thiolated probe, and MCH, respectively. It has been shown that a high response is obtained with the intercalative behavior of OB after hybridization between the probe DNA and the target microRNA in this layer. The detection limit was calculated as 0.6 fM, with the target miRNA ranging from 2.0 fM to 8.0 pM. The designed intercalator-based nanobiosensor was able to clearly distinguish single, tri-base mismatch and non-complementary miRNAs from complementary target miRNA. 

An important study that should not be overlooked for cancer detection through the use of microRNAs was carried out by Tran et al. [87] for the determination of infarctus biomarker and prostate cancer biomarker miR-29b-1 and miR-141, respectively. In this biosensor design, both conductive polymer and graphene oxide were used in the modification of the glassy carbon electrode surface, and the detection limit could be reduced to 5 fM, depending on the surface area increase of the nanomaterial structure. In the study, the probe DNA was covalently attached to the glassy carbon electrode and then hybridized with microRNA sequences under appropriate conditions. Then, the incubation process, which was carried out under suitable conditions for the complexation reaction of the hybrid structure and the special antibodies, recognizing miRNA−DNA heteroduplexes, was performed. Finally, electrochemical measurements were made with cyclic voltammetry and square wave voltammetry techniques to monitor the relevant bindings on the sensor surface. In their design, the purpose of using antibodies is to double-check the selectivity of the developed biosensor system. 

In the detection strategy of the analyte; when only probe DNA is present on the surface, low current response is observed, increased current response after hybridization and decreased current response again after complexation with antibody were obtained, so the determination was carried out in a controlled manner. Then, when the RNA/DNA hybrids were reintroduced into the solution, an increase in the current signal was obtained again (current decrease is expressed as signal off, current increase is expressed as signal on). In this system, the selectivity specific to the target analyte is double-checked. In addition, the complexation of antibodies on the electrode surface is a mechanism confirming the presence of miRNA. It is important that this On–Off–On detection strategy is used as a “triple-validation system” to increase the reliability of the results. At this point, the necessity of taking this study as a case study by scientists for new biosensor designs should be evaluated in terms of increasing the quality of the designs. After this study, a different immunosensor design with screen-printed gold electrode modified with reduced graphene oxide and carbon nanotubes, carried out by the same research group the following year, also stands out for the determination of microRNA-based cancer biomarkers [41].

Another prominent design developed for the diagnosis of breast cancer-related gene (BRCA 1) was developed by Rasheed et al., [29] based on measurement of the electrochemical signal of gold nanoparticle. Surface modification of the biosensor using graphene oxide-modified glassy carbon electrode was performed by immobilization of capture probe DNA, target DNA and signaling probe DNA, respectively. Since the signaling probe carries a gold nanoparticle label, the electrochemical signal resulting from the oxidation of the gold at 1.1V was obtained by using cyclic voltammetry (CV) in the presence of the target analyte when the sandwich hybridization event occurred. The biosensor performance was also evaluated chronoamperometry. The limit of detection of the developed biodevice was calculated as 1 fM (5.896 femtogram/mL).

An innovative and remarkable biosensor platform developed for cancer prognosis has been reported by Elshafey et al., where gold nanoparticles provide large surface area for p53 protein modification and reduced graphene oxide is used for signal enrichment [88]. In the study, the [Fe(CN)_6_]^3−/4−^ redox couple was used in measurements to monitor both surface modifications and antibody-antigen binding via CV or SWV methodologies. In addition, the electrochemical signal of gold nanoparticle label was also evaluated in the determination. In this system, p53 antibodies could be detected with a detection limit of 0.088 pg/mL with a linear range of 0.1 pg/mL to 10 ng/mL.

**Figure 5:**
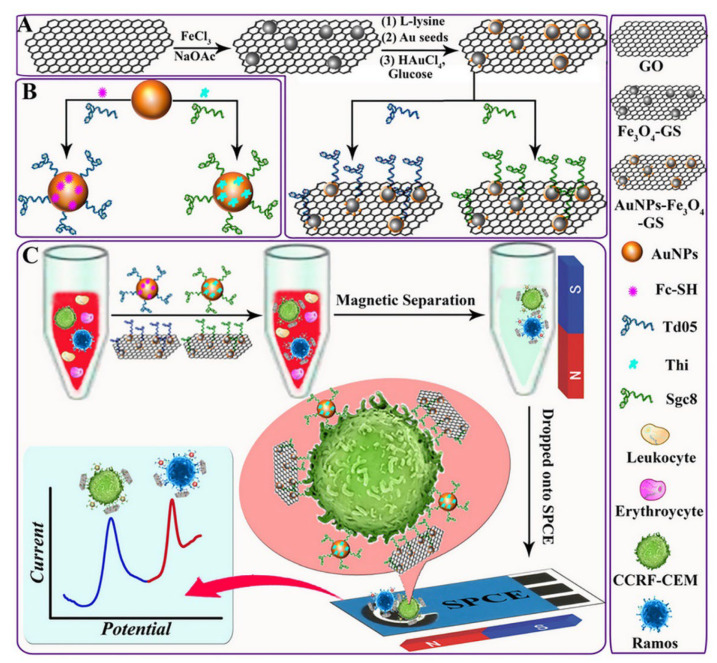
Schematic representation of the synthesis of (**A**) the aptamer-carrying “AuNPs-Fe_3_O_4_-graphene sheet” based bionanocomposite capture probes and (**B**) the aptamer/electroactive substances-modified AuNPs Signaling Probes and (**C**) detection protocol of the developed biosensor containing capture, magnetic separation, signal amplification and target sensing steps. Reprinted with permission from Ref. [89]. Copyright 2019, American Chemical Society.

Dou et al. [89] demonstrated an aptamer-, magnetic bead-, gold nanoparticle-, and graphene oxide-based nanobiosensor for the detection of circulating tumor cells (CTCs). In this work, the determination of trace amounts of CTCs in whole human blood was the objective, and for their detection, a “new capture probe” structure was designed with a magnetic graphene nanolayer decorated with an aptamer and gold nanoparticle layer (AuNP). Apart from this, an aptamer structure containing electroactive species (ferrocene or thionine)-loaded AuNPs was also prepared and used as signal amplification probe. Incubation of both probes (capture and signaling probes) with sample solutions containing target CTCs and subsequent magnetic separation were then performed. After that, the signals of the electroactive species were measured using the screen-printed electrode as a function of the binding aptamers and cancer cells. Electrochemical signals originating from the ferrocene and thionine were obtained at two different potentials and multiple measurements could be taken with the square wave voltammetry (SWV) technique (Figure 5). In the study, two different target hematological tumor cells (CTCs) were analyzed with detection limits down to 4 and 3 cells/mL by using two different aptamer sequences simultaneously in whole blood by spiking CTCs into the real sample. 

On the other hand, in paper-based biosensors, another sensor platform, graphene oxide nanomaterial and various labels are used for signal amplification and enrichment of the sensing surface area in the diagnosis. In fact, biosensors based on GO-modified paper have been produced since the 2010s. Electrochemical biosensor designs in cancer diagnosis have also been a prominent research area in recent years due to paper-based electrodes. Nevertheless, it is still considered that there are some issues that need to be overcome in order to reveal point-of-care systems [90]. A few interesting recent studies will be presented here. 

**Figure 6:**
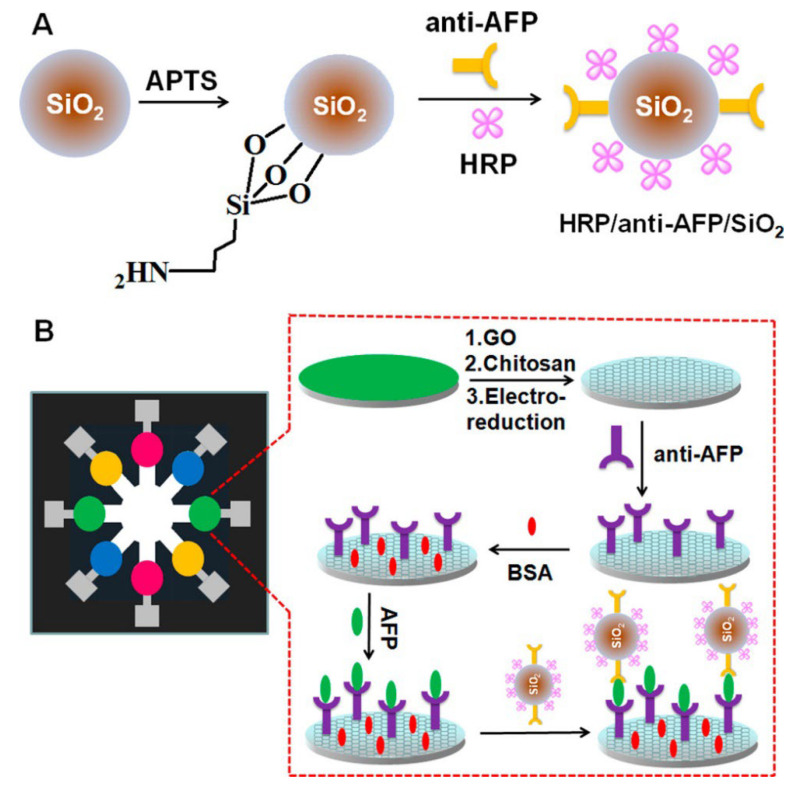
(**A**) HRP and Antibody modification on SiO_2_ Nanoparticle surface. (**B**) Microfluidic Paper sensor structure and biomodification processes on the graphene oxide-based electrode surface in the sensor. Reprinted with permission from Ref. [91]. Copyright 2013, American Chemical Society.

A remarkable case study in this area was demonstrated by Wu et al. [91] for ultrasensitive multiplexed detection of cancer biomarkers based on a paper-based microfluidic electrochemical immunosensor containing graphene film. In the developed device, graphene oxide was used to accelerate electron transfer, and signal enrichment was also provided on the surface using silica nanoparticles (SiO_2_) to further increase the sensor performance. The paper-based part in the microfluidic paper-based analytical device (μPAD) consists of two layers of selectively patterned square filter paper of the same size (35.0 mm × 35.0 mm), and the specially patterned parts, where the analysis will be carried out, were designed with the AutoCAD 2012 program. Eight carbon-based circular working electrode regions are printed on the paper surface using screen-printed technology (Figure 6). In the experimental steps for analysis, antibodies (four types) and horseradish peroxidase (HRP) were first immobilized on silica particles by surface chemistry protocols. Then, graphene oxide and chitosan modification and then electroreduction processes were applied to the working electrode on the paper surface. Capturing antibodies were attached to the prepared surface by chemical treatment, and, after washing and blocking, this immunodevice was stored in the refrigerator until used. For electrochemical measurements, solutions containing antigens (AFP, CEA, cancer antigen125 (CA125), and carbohydrate antigen 153 (CA153) were added to each working electrode and incubation was provided first. Then, the interaction of these antigens with HRP-carrying silica nanoparticles prepared by pre-modification was provided. After this interaction, if antigen–antibody binding occurs, the electroactive product 2,2′-diaminoazobenzene is formed as a result of the enzymatic reaction with O-phenylenediamine and H_2_O_2_ added to the medium, and voltammetric analysis can be performed by this means. The developed method was able to reach the detection limit of pg/mL and this was evaluated as being successful in clinical use. 

The following year, the same research group developed a new sensor that can detect carcinoembryonic antigen (CEA), alpha fetoprotein (AFP), cancer antigen 125 (CA125), and carbohydrate antigen 153 (CA153) with 0.01, 0.01, 0.05, and 0.05 ng/mL of detection limits respectively. In that design, they performed sensitive determination of relevant cancer biomarkers using (i) polymerization-assisted signal amplification, (ii) HRP-O-phenylenediamine-H_2_O_2_ electrochemical detection strategy, (iii) sandwich assay protocol, and (iv) a microfluidic system [92].

**Figure 7:**
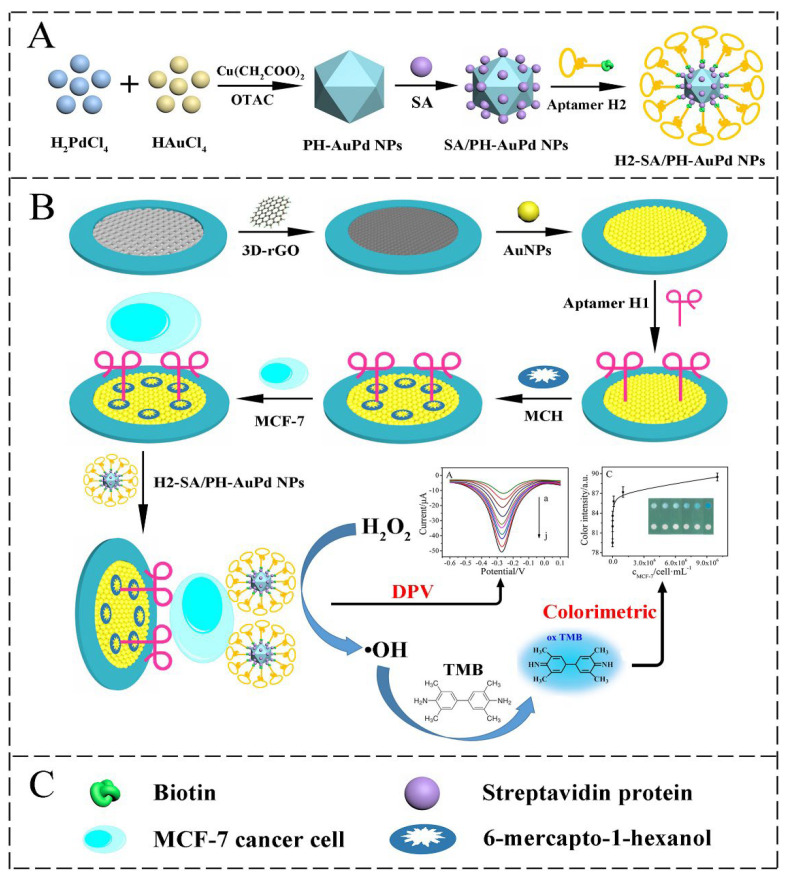
Modification stages of the working electrode in the paper-based biosensor system. (**A**) Preparation of the aptamer-streptavidin-polyhedral AuPd alloy nanoparticle structure (H1-SA/PH-AuPd NPs) (**B**) the total analysis procedure for MCF-7 cells. Reprinted with permission from Ref. [93]. Copyright 2018, Elsevier.

As the schematic detection procedure is shown in Figure 7, Wang et al. [93] carried out the determination of breast cancer MCF-7 cells with a paper electrode (Au@3D-rGO/PWE), which they prepared by modifying gold nanoparticles on the three-dimensional reduced graphene oxide (3D-rGO) surface, at the detection limit of 20 cells/mL. Their cytosensor design included polyhedral AuPd alloy nanoparticles (PH-AuPd NP) as catalytic material and two different aptamer sequences for cancer cell capturing. In the study, this Pd-based nanoparticle was prepared by synthesis and modified with one of the aptamer sequences (Aptamer-H2). The other aptamer was immobilized on the paper working electrode surface after 3D-rGO and AuNPs modifications. When MCF-7 cells were detected on the paper-based electrode, PH-AuPd NPs were also attached to the surface of the cells thanks to the second aptamer they carried, and thus an amplified electrochemical response was obtained by catalyzing H_2_O_2_ for •OH production. As a result of this electrochemical reaction, a huge signal was obtained. For the main structure, size and shape of the lab-on-paper device please see the reference [93]. In the study, determination on the basis of colorimetry was also performed, and it was evaluated that this dual-mode cytosensor design is applicable for clinical applications and mobile health care.

**Figure 8:**
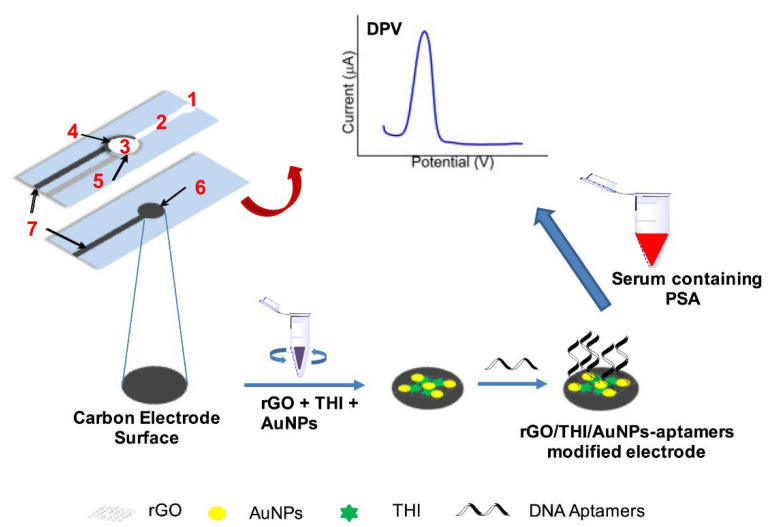
Diagram showing the fabrication and analysis steps of the microfluidic paper-based aptasensor device. (1) Injection site, (2) microfluidic channel, (3) reaction zone, (4) screen-printed carbon counter electrode, (5) screen-printed reference electrodes, (6) screen-printed working electrode; (7) the tip of the screen-printed electrode. Reprinted with permission from Ref. [94]. Copyright 2018, Elsevier.

Biosensor approaches containing graphene oxide, gold nanoparticles, and thionine have also been preferred by different research groups and have been used since the 2010s for cancer diagnosis [44]. For example, a sensitive platform using microfluidic paper electrode containing reduced graphene oxide (rGO), thionine, and gold nanoparticles and where Prostate-specific antigen (PSA) analysis has been recently performed from real serum samples based on aptamer–antigen binding strategy by Wei et al. [94]. In the paper-based biosensor device, the hydrophobic and hydrophilic layers of the microfluidic channel were prepared by wax-printing, and then the structure of the triple electrode system was formed on the paper surface via screen printing technology (Figure 8). In the design, in which reduced graphene oxide and gold nanoparticles were used both to enrich the surface area and to increase the conductivity, thionine (THI) was also included as an electrochemical indicator molecule (AuNPs/rGO/THI nanocomposites). The basis of the determination is based on monitoring the gradual decrease in voltammetric THI response with increasing antigen concentrations. With the developed methodology, analyses were also performed on serum samples and the limit of detection (LOD) was calculated as 10 pg/mL, in the dynamic range of 0.05 ng/mL to 200 ng/mL (R^2^ = 0.995).

Fan et al. reported another paper-based study in the cancer diagnosis field for detection of cancer antigen 125 (CA125) based on antibody–antigen interaction, including thionine, gold nanoparticles, and rGO with the limit of detection (LOD) of 0.01 U/mL [95]. 

**Figure 9:**
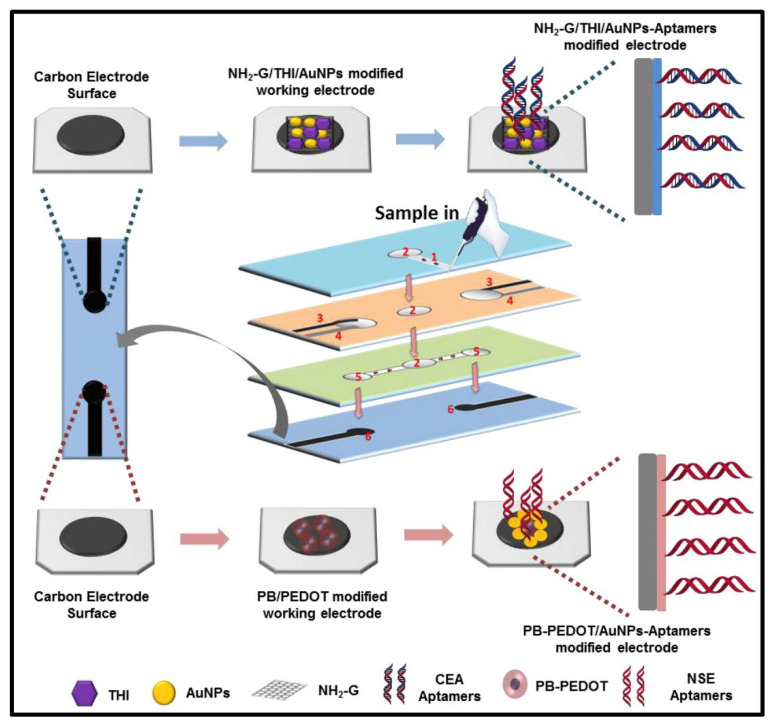
Construction of the electrochemical paper-based aptasensor device; (1) sample inlet area (2) filter (3) screen-printed counter electrode; (4) screen-printed reference electrode; (5) detection zone; (6) screen-printed working electrode. Reprinted with permission from Ref. [96]. Copyright 2019, Elsevier.

Cai’s group followed an interesting paper-based strategy in 2019 for simultaneous detection of tumor biomarkers called carcinoembryonic antigen (CEA) and neuron-specific enolase (NSE) in clinical samples [96]. In this study, firstly, two different nanocomposite structures (Amino functional graphene (NG)–Thionine (THI)–gold nanoparticles (AuNPs) and Prussian blue (PB)-poly(3,4-ethylenedioxythiophene) (PEDOT)- AuNPs) were synthesized. These nanocomposites were then individually coated on working carbon electrodes on the microfluidic paper surface. Then, after each of the thiol-labeled aptamers were immobilized to two different working electrodes, high electrochemical signals originating from thionine and Prussian blue were obtained. After the aptamer–antigen binding, a decrease in these signals was observed and thus the analysis was performed on the basis of the “signal decrease” strategy, with the detection limit of 2 pg/mL for CEA and 10 pg/mL for NSE, respectively (Figure 9). This study distinguishes itself when compared to other paper-based biosensors studies in the literature in terms of directly analyzing clinical serum samples and presenting the results in comparison with the standard method.

**Figure 10:**
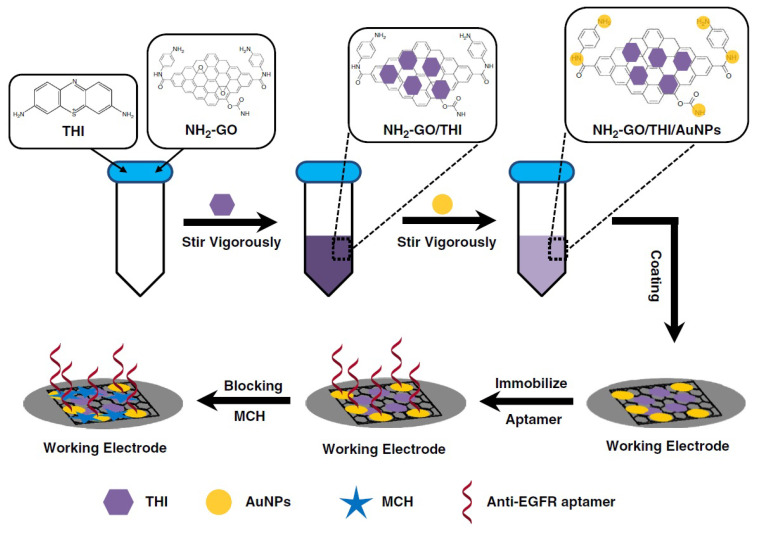
The construction of the origami paper-based aptasensor surface. Reprinted with permission from Ref. [97]. Copyright 2020, this article is licensed under a Creative Commons Attribution 4.0 International License.

An origami paper-based electrochemical aptasensor design study for the detection of overexpressed epidermal growth factor receptor (EGFR) in the presence of various carcinomas was performed by Wang et al. [97] The preparation of the sensor surface consists of some steps such as synthesis of amino-functional graphene (NH_2_-GO)/thionine (THI)/gold particle (AuNP) nanocomposites, and modification to the carbon working electrode, covalent coupling of aptamers to the modified transducer, and surface blocking with mercaptoethanol (Figure 10). The performance of the developed origami paper-containing aptasensor was tested using standard buffer solutions and spiked with different concentrations of EGFR into the serum sample. Binding of EGFR aptamer and antigen was determined by CV and DPV measurements (based on the THI signal decrease), and it was shown that the aptasensor could reach the detection limit of 5 pg/mL for EGFR in the linear range of 0.05 to 200 ng/mL (R^2^ = 0.989). Advantages of the system developed include precise analysis with low sample volume and ease of use of the device. In addition, it has been proven that the working electrode modification with graphene-based nanocomposite increases the electron transfer efficiency by enhancing the detection signal.

Ortega et al. [98] developed a paper-based immunosensor for the detection of novel epithelial biomarkers for breast cancer, Claudin 7, and CD81, in circulating extracellular vesicles (EVs). The sensing surface of the developed dual electrochemical immunosensor device includes electrodes printed with inks containing graphene oxide and silver powders, and the sensor works according to the sandwich-type sensing methodology. There is a modified Claudin 7 antibody in one of the fluidic paper channels of the immunosensor and a CD81 antibody in the other channel. Then, these antibodies were incubated with antigens, which are related cancer biomarkers. After this process, binding was performed with secondary antibodies containing a mixture of HRP-anti-Claudin 7 and HRP-anti-CD81 (HRP-labeled antibodies), and analysis was performed on the basis of the enzymatic reaction resulting from this interaction. In this reaction, 4-tert butyl catechol was used as the substrate and the amperometric signal of the enzymatic product p-benzoquinone formed in the presence of H_2_O_2_ was measured. The detection limits and linear range for the developed immunosensor were found to be 0.4 pg/mL (2 to 1000 pg/mL) and 3 pg/mL (0.01 to 10 ng/mL) for Claudin 7 and CD 81, respectively. Another interesting aspect of this study is that it has higher diagnostic accuracy than traditional biomarker methods by working with samples taken from 60 breast cancer patients and 20 healthy volunteers.

**Figure 11:**
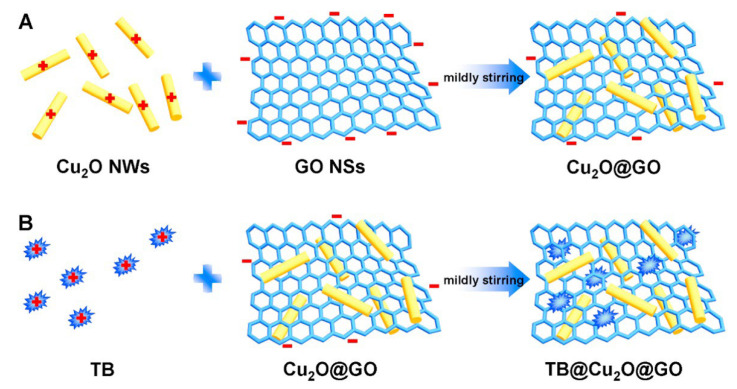
Two-step preparation (A and B) of TB@Cu_2_O@GO composite nanomaterial. Reprinted with permission from Ref. [99]. Copyright 2017, Elsevier.

One of immunosensor studies was carried out by Wang et al. [99] on the basis of cuprous oxide nanowire-decorated graphene oxide nanosheet nanocomposites for the detection of tumor marker “alpha fetoprotein (AFP)”. In the study, first of all, TB@Cu_2_O@GO nanocomposite preparation processes were carried out. For this purpose, graphene nanosheets (GO NSs) and copper nanowires (Cu_2_O NWs) were interacted with each other under suitable conditions, and then, by adding toluidine blue to the reaction medium, the targeted composite structure was formed (TB@Cu_2_O@GO nanocomposites, Figure 11). Then, antibody (anti-AFP) was attached covalently on this composite structure, which was modified on the glass carbon electrode surface. In this device, toluidine blue was used as an electron transfer mediator after adsorbing to the composite structure, and a decrease in the voltammetric response of TB was observed as a result of antibody–antigen binding. The detection limit for this biosensor was calculated as 0.1 fg/mL.

There is also much literature in which indicator dyes are immobilized on the working electrode surface and analysis is carried out thanks to the electrochemical signal of the related molecule. For example, there is one interesting biosensor design developed by Gao et al., in which carcinoembryonic antigen (CEA) analysis is performed under the indicator of the Nile blue (NB) substance [100]. A biosensor, in which hemin-functionalized graphene-conjugated palladium nanocomposite is used in the design and the determination is made through hemin signal measurement, is also available in the literature [101]. 

Another electroactive dye-assisted nanobiosensor architecture was proposed by Pothipor et al. [102] for the simultaneous analysis of breast cancer biomarkers (miRNA-21, miRNA-155, and miRNA-210) in which anthraquinone (AQ), and polydopamine (PDA) are used as redox molecules, together with methylene blue (MB), has also recently taken its place in the literature. Looking at the content of the design, it is seen that there is a triple screen-printed carbon working electrode (3SPCE) with an array-shaped structure modified with electroactive dye/gold nanoparticles/graphene quantum dots/graphene oxide (dye/AuNPs/GQDs/GO) composite. Electrochemical measurements with these multiple electrodes were performed using a common reference and a counter electrode. First, relevant capture miRNA probes were attached to each working electrode surface via SH–Au interaction, and then hybridization was carried out with target miRNAs under appropriate conditions. The decrease in the responses obtained from the redox probes modified on the electrode surface after hybridization formed the basis of the determination. The linear range of the biosensor for microRNA analysis is between 0.001 and 1000 pM, with LODs of 0.04, 0.33, and 0.28 fM for miRNA-155, miRNA-210, and miRNA-21, respectively. In addition, the performance of the developed biosensor was demonstrated in 50% diluted human serum samples, and satisfactory results were achieved.

**Figure 12:**
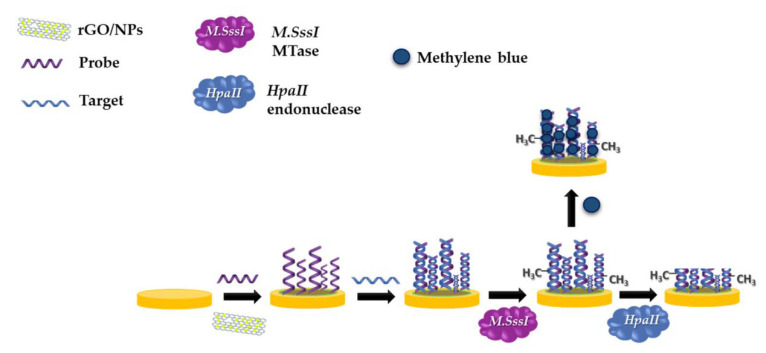
Schematic illustration of DNA biosensor construction and detection process. Reprinted with permission from Ref. [103]. Copyright 2020, this article is licensed under a Creative Commons Attribution 4.0 International License.

Sedlackova et al. [103] developed a biosensor containing rGO with a DNA hybridization strategy, based on the fact that the methyl groups of tumor cells show an impaired DNA methylation pattern compared to normal cells. In the methylation analysis performed by applying a different approach according to the existing biosensor technologies, to further increase the gold electrode surface area, rGO decorated with three different nanoparticles, those being gold (AuNPs), silver (AgNPs), and copper (CuNPs), was used. First, ssDNA was immobilized on each prepared surface and then the modified surface was treated with target DNA for hybridization. Then, CpG (50-CCGG-30) islands formed by methylation with M.SssI methyltransferase (MTase) were cut with a specific endonuclease enzyme (Figure 12). The methylation determination was performed using differential pulse voltammetry (DPV) through electroactive methylene blue indicator. In addition, characterization of the biosensor surface was performed by the electrochemical impedance spectroscopy method. The detection limit (LOD) of the developed biosensor was found to be 0.06 U/mL for synthetic DNA sequences and 0.19 U/mL for real serum sample. In line with the results obtained, it has been evaluated that this system developed for methylation analysis has the infrastructure to be used in clinical applications.

An important electrochemical GO-based biosensor with features that can be used for point-of-care applications (POCT) has been recently reported for prostate-specific antigen (PSA) determination by Pothipor et al. [104] In the study, first graphene-poly(3-aminobenzoic acid) (GP-P3ABA) nanomaterial-conductive polymer composite modification was made on the screen-printed carbon electrode surface by polymerization. For signal amplification, metal-ion-loaded porous-hollowed-silver-gold core-shell nanoparticles (PHSGNPs) were then synthesized. In this immunosensor, in which sandwich detection technique is preferred, primary antibody immobilization using carbodiimide chemistry on the GP-P3ABA modified electrode surface, BSA surface blocking, antigen incubation, labeling with metal ion(Cu^2+^)-loaded nanoparticles (PHSGNPs or AuNPs) carrying secondary antibody and electrochemical monitoring of binding event steps were applied, respectively. Thanks to both the electrode surface being covered with graphene-containing composite and the ability to connect a large quantity of probes to the pores of this structure, the signal obtained was increased by 16 times compared to those without composites. In order to evaluate the performance of the developed sensor system, the use of the label containing PHSGNP and the label containing only gold nanoparticles were compared. In the assay performed using PHSGNP, the detection limit was obtained approximately 120 times lower than the other (0.13 pg/mL). In order to measure the usability of this system in clinical applications, PSA was also spiked into human serum and successful results were obtained.

**Figure 13:**
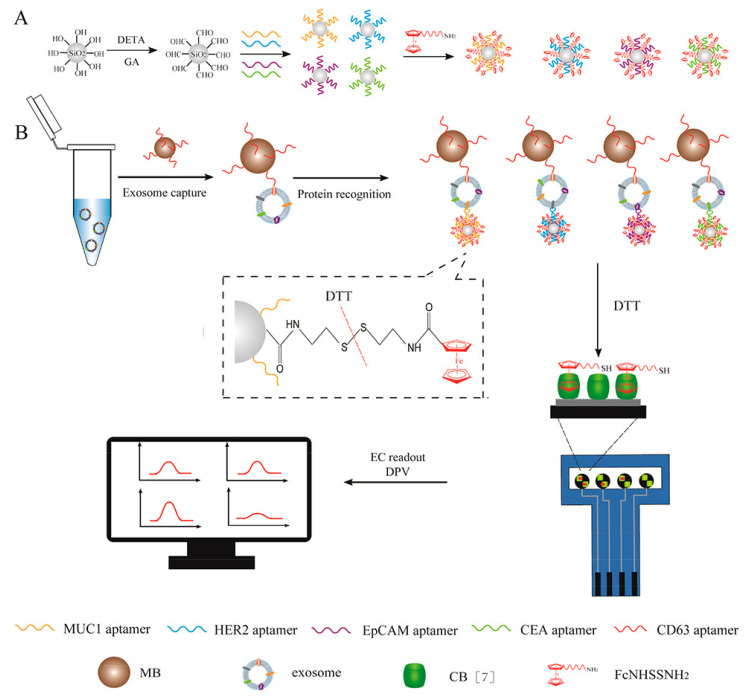
Schematic illustration of the magneto-mediated Electrochemical Sensor. (**A**) The preparation of “signaling probe” structures containing silica nanomaterials (SiO_2_ NPs) modified with MUC1, HER2, EpCAM, or CEA aptamers and ferrocene.; (**B**) Schematic illustration of the exosome analysis protocol performed between CD63 protein-specific aptamer-modified magnetic beads (MB) and the signaling probe. Reprinted with permission from Ref. [105]. Copyright 2020, American Chemical Society.

A highly applicable and innovative design for clinical diagnosis in the biosensors area using magnetic beads and graphene oxide, was developed by An et al. in 2020 [105] for the simultaneous and combined analysis of breast cancer exosome proteins. In the study, CD63 protein-specific aptamer, which is expressed on the surface of many breast cancer exosomes, was first modified on the surface of magnetic beads (MBs) and used as a probe to capture exosomes. On the other hand, silica nanomaterials (SiO_2_ NPs) were individually modified with MUC1, HER2, EpCAM, and CEA aptamers to detect their specific exosomes (Figure 13). These aptamers were also used in the analysis as labeled with an electrochemical label of ferrocene origin. After the capture of exosomes, the sandwich-binding protocol is completed as a result of the interaction of silica nanoparticles carrying specific aptamers and MBs. After magnetic separation, using dithiothreitol (DTT), the indicator redox molecule was released from silica particles and taken into the supernatant. Next, the voltammetric peak of ferrocene oxidation was measured using a screen-printed carbon electrode (SPCE) modified with graphene oxide-cucurbit [7] (GO-CB [7]) prepared before the experiment. When the exosome is found at a concentration of 1.2 × 10^6^ particles/μL in the sample, the analytical signal increases approximately 7-fold compared to the experimental conditions without the exosome. This shows that magnetic beads and graphene-based electrochemical sensor can sensitively perform exosome analysis. Accurate analyses were also performed on real serum samples from breast cancer patients and healthy individuals using the developed detection strategy, demonstrating the strong potential of the system in clinical diagnosis.

#### 1.4.2. Label-Free Electrochemical Biosensors

##### Label-Free FET-Based Biosensors

A semiconductor device with three terminals named source, drain, and gate that uses the electric field to control the current in the device is called Field Effect Transistor (FET). Graphene oxide can also be used in the design of this device (GFET) and in this way, channel conductivity can be changed, as well as various capture biomolecules can be modified on the graphene material surface.

Aspermair et al. [106] designed a reduced graphene oxide field-effect transistor (rGO-FETs)-based biosensor for the detection of human papillomavirus (HPV), which is considered a cervical cancer biomarker. In this study, RNA aptamer specific to the relevant cancer type was immobilized on the FET surface to detect HPV-16 E7 protein, which is the target analyte. The aptamer-modified rGO-FET device demonstrates aptamer-protein binding in real time and label-free, and the presence of the target protein is evidenced by a large increase of obtained response as a result of binding. The detection limit of HPV-16 E7 protein for this FET sensor was found to be approximately 100 pg/mL (1.75 nM), with a linear range of 30 and 1000 nM. Moreover, to test the validity of the developed rGO-FET device in clinical applications, saliva samples with different concentrations of protein were also studied. The compatibility of the data obtained in both the buffer and saliva test environment showed the success of the developed system.

On the other hand, technologies that bring a new perspective to evaluate all processes in cells together have attracted a lot of attention in recent years. In this area, designs that provide analysis of microvesicles (MVs) come to the fore. Microvesicles are structures involved in intercellular communication, and nearly all cells (including cancer cells) release MVs to communicate with other specific recipient cells. In this respect, analyzing the difference of amount before and after the release of MVs is important in diagnosis in the field of medicine.

A FET biosensor was developed by Wu et al. to overcome the current difficulties in sensitive detection of MVs. Reduced graphene oxide and streptavidin were used in the FET surface design (SA-functionalized FET biosensor). For biosensing, MVs were biotinylated (B-MV) via a membrane biotinylation strategy developed in this work. The detection limit of the FET biosensor, which can specifically recognize B-MVs due to the high affinity between SA and biotin, is 20 particles per µL. The developed device separated B-MVs from other non-biotinized structures. It also has the ability to detect biotinylated B-MVs of interest by targeting different cells, including cancer cells and normal cells [107].

**Figure 14:**
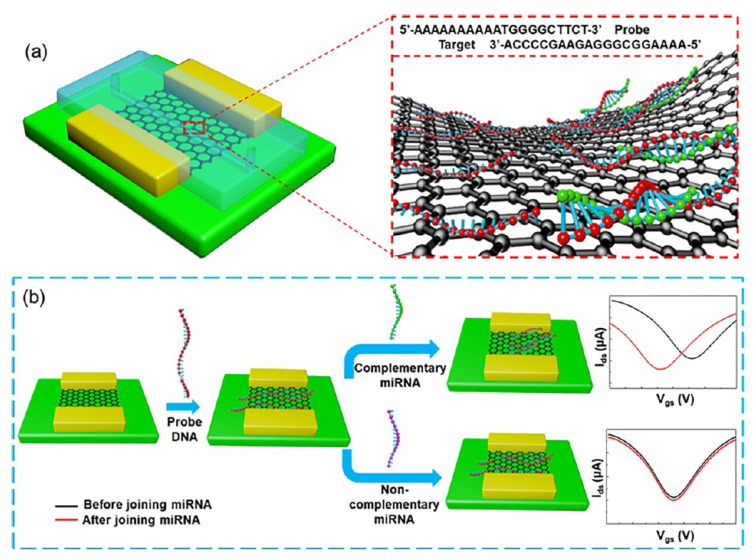
(**a**) The surface structure and (**b**) the detection principle of the miRNA FET biosensor. Reprinted with permission from Ref. [108]. Copyright 2020, American Chemical Society.

Another FET-based innovative approach in cancer diagnosis in combination with microfluidic chips has also been recently reported by Gao et al. [108] In the study, microRNA (miR-4484)-based breast cancer detection was performed with a flexible graphene field-effect transistor (Gr-FET) biosensor. In the determination, after the modification of the FET surface with graphene, first immobilization of the probe DNA and then hybridization with target or non-specific miRNA sequences were performed. Interestingly, only a certain part of the probe and target sequences are complementary to each other. In this way, a clear response difference (dirac point difference) was obtained with the FET system before and after hybridization (Figure 14). It is a great advantage that the developed system is label-free and non-functionalized, and it is stated that the flexible biosensor offers good performance even if there are many bending situations. The detection limit of the Gr-FET based biosensor is 10 fM and the biosensor reaches this result in 20 min. The experimental results were also confirmed by the RAMAN spectroscopy technique.

##### Label-Free Amperometric/Potentiometric Biosensors

Gu et al. has reported light-addressable potentiometric sensor (LAPS) for the label-free detection of circulating tumor cells (CTCs) of prostate cancer by using mouse anti-human epithelial cell adhesion molecule (anti-EpCAM) monoclonal antibody as the capture probe which was immobilized to the sensor surface containing graphene oxide modified with carboxylic acid group (GO-COOH) [109]. LAPS chips were silanized by APTES molecule first and GO-COOH was grafted on the pretreated surface. Then carbodiimide chemistry was applied to graphene oxide-covered sensor followed by the anti-EpCAM antibody immobilization and surface blocking with bovine serum albumin. Bio-functionalization of LAPS took nearly 24 h. Antibody-cell binding experiments were performed on three different cancer cell lines, and signals obtained from the LAPS chip were followed for the determination of these CTCs. As a result of the experiment, a gradual decrease in LAPS voltage signal was observed after binding with increasing antigen cell number. It was stated in the study that the decreasing LAPS output voltage was caused by the impedance of the CTCs attached to the sensor surface. It is predicted that the developed potentiometric detection system can also analyze different cancer cells if specific antigen-antibody selections are made for each assay. The acceptable limit of detection for the GO-COOH LAPS platform was calculated as 10 CTCs in 1ml of blood sample. However, this method needs further development in order to overcome the interference effects in cases where miRNA, protein, and dead carcinoma cells are found in the analysis sample. In addition, in this study, immunofluorescence experiments (IFA) were also performed in parallel with the LAPS analysis, and the validation of developed methodology was provided for CTC detection.

**Figure 15:**
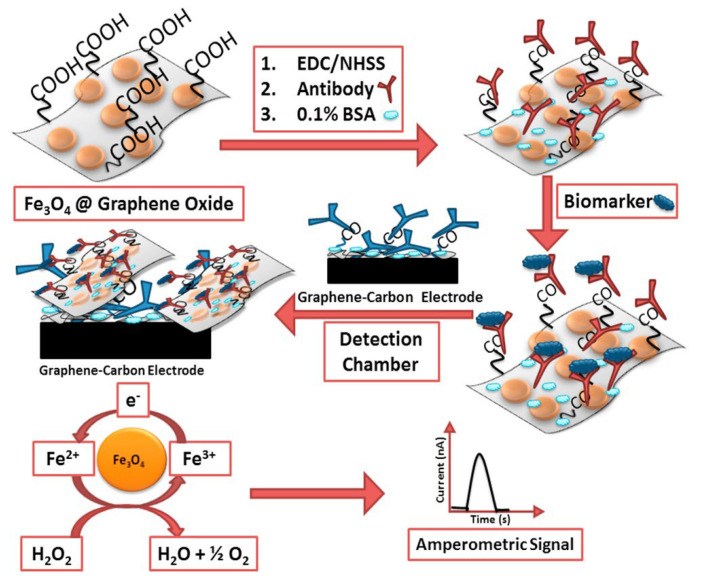
Schematic illustration of the experimental protocol including the preparation of paramagnetic composite (Fe_3_O_4_@GO), immobilization of detection antibodies on the composite surface, blocking with BSA, binding of antigen biomarker with antibody, interaction between the modified composite and electrochemically reduced graphene oxide (ERGO)-carrying secondary antibody-coated electrode, and measurement by using amperometry. Reprinted with permission from Ref. [110]. Copyright 2017, Elsevier.

In another work, Sharafeldin et al. [110] demonstrated that a label-free amperometry-based detection strategy for the clarification of biomarker proteins prostate-specific antigen (PSA) and prostate-specific membrane antigen (PSMA). For the ultrasensitive determination, firstly, the paramagnetic composite structure (Fe_3_O_4_@GO, 1 μm size range) was formed by adding Fe_3_O_4_ nanoparticles to graphene oxide (GO) nanolayers. Then, by immobilizing detection antibodies specific to PSA and PMSA biomarkers on the composite surface and then binding the relevant antigens this structure gained the “detection probe” feature. The surface of eight-sensor screen-printed carbon arrays in the microfluidic system used for detection was first coated with electrochemically reduced graphene oxide. A second set of antibodies was then modified to the electrode surface, creating the “capture probe” section in the sensor. After incubation of Fe_3_O_4_@GO nanocomposite particles carrying biomarkers with secondary antibodies (second set of antibodies), sandwich-type biomolecule binding on the electrode surface was achieved, and amperometric determination was performed by adding H_2_O_2_ to the medium (Figure 15). Here, the amounts of Fe_3_O_4_@GO bound to the sensor are proportional to the specific protein concentrations, and the reaction is due to the decomposition of H_2_O_2_ catalyzed by Fe_3_O_4_ nanoparticles. In the study, the detection limits of PSA and PSMA were calculated as 15 fg/mL and 4.8 fg/mL, respectively, with only $0.85 of reagent cost for a single two-protein assay.

In this area, there are also biosensors in which poly 3,4-ethylenedioxythiophene (PEDOT) is used. For example, a biosensor design based on a paper electrode and using poly (3,4-ethylenedioxythiophene): poly(styrenesulfonate) (PEDOT:PSS) and rGO composite was made by Kumar et al. [111] for cancer biomarker (carcinoembryonic antigen, CEA)-detection from cancer patient serum samples in the linear range of 1–10 ng/mL. In the system in which the antibody is immobilized by physical adsorption and the binding between antigen and the related Ab is determined both impedimetric and amperometric techniques, a sensitivity of 25.8 mA ng^−1^ mL cm^−2^ was obtained.

In another PEDOT: PSS-based and aptamer-containing study, the determination of carcinoembryonic antigen (CEA) was carried out impedimetrically in standard buffer solution and human serum samples, with a detection limit of 0.45 ng/mL and 1.06 ng/mL, respectively [112]. Although this study is based on EIS, it is included in this section because it contains PEDOT.

**Figure 16:**
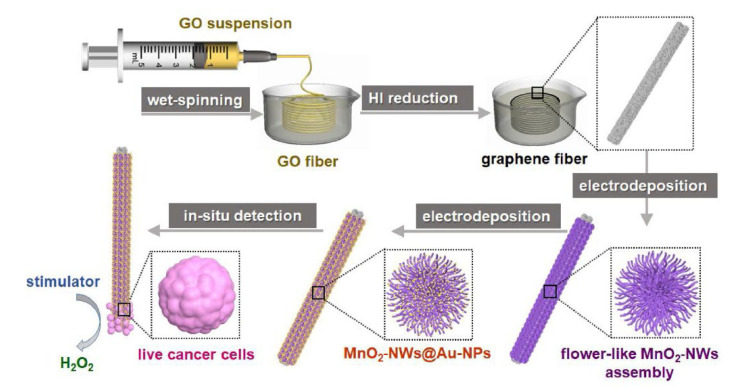
The fabrication steps of MnO_2_-NWs@Au-NPs/GF microelectrode for the detection of live cells. Reprinted with permission from Ref. [113]. Copyright 2020, Elsevier.

Another prominent development in biosensors developed in this field is the measurement of hydrogen peroxide released from cancerous cells and thus detection of the presence of cancer. Zhao et al. developed an amperometry-based electrochemical biosensor and made the diagnosis of cancer by measuring the hydrogen peroxide released from living cancer cells. For this application, they designed a nanohybrid microelectrode-containing graphene fiber (GF) modified with double nanoenzyme MnO_2_-NWs@Au-NPs and prepared with the graphene oxide (GO) starting material of the synthesis [113] (Figure 16). The preparation of Au nanoparticles (Au-NPs) wrapped in flower-like MnO_2_ nanowires (MnO_2_-NWs) was firstly carried out for modification of the fiber coating the microelectrode surface. For this immobilization, a wet-spinning method was preferred by the researchers, and with this dual modification (MnO_2_-NWs@Au-NPs) formed on the fiber surface, enzyme-mimicking catalytic nanomaterial activity is obtained. In fact, this nanoenzyme technology has shown great promise in many fields (medicine, chemistry, food, environment, etc.) since its discovery on Fe_3_O_4_ nanoparticles in 2007 [114], thanks to its advantages, such as low cost, ease of production, and high catalytic activity etc. [115,116]. In the study [113], the superior catalytic activity of the microelectrode with a dual nanoenzyme structure was used to provide the basic redox reaction of H_2_O_2_ with high efficiency. In this way, real-time analysis of H_2_O_2_ released from human breast cancer cells with high sensitivity and selectivity could be performed amperometrically. With the developed MnO_2_-NWs@Au-NPs/GF microelectrode, a high sensitivity of 32.9 μA cm^−2^ m^−1^ and detection limit of 1.9 μM could be reached in the linear range of 0.01~9.51 mM. It was stated that the developed sensor was used not only to distinguish cancer cells from normal cells, but also to identify different cancer cells, and it was determined that the system could also be used in on-site analysis.

In another study, an intrinsic peroxidase-like activity-based biosensor involving copper nanoparticles was performed using O-phenylenediamine (OPD) substrate and H_2_O_2_. In the study, Alizadeh et al. [117] used a graphene oxide nanosheet decorated with CuO/WO_3_ nanoparticle (CuO/WO_3_-GO) for the detection of cancer cells. In order to prepare the material to be coated on the sensor surface, CuO/WO_3_-GO synthesis was carried out on GO layers with the reaction carried out in an alkaline environment. Then, folic acid was bonded to this nanocomposite structure by surface chemistry techniques, thus, the “detection probe” of the developed biosensor was prepared. For the electrochemical determination of the cancer cell, a 96-well plate was used. Different numbers of cells were fixed at room temperature by placing them on this plate. Afterwards, the CuO/WO_3_-GO nanocomposite structure was added on them and left for incubation. After washing, an intrinsic peroxidase-like reaction occurred with the addition of buffer containing OPD and H_2_O_2_. In this strategy, a high electrochemical response was obtained as a result of the efficient enzymatic reaction in the absence of cancer cells, while a very low signal was obtained as a result of the removal of some OPD-H_2_O_2_ from the electrode by participating in the chemical reaction in the presence of cells. With the developed methodology, the detection range was determined to be 50 to 105 cells/mL and the detection limit was determined as 18 cells/mL.

Another biosensor in this area has been developed for the determination of hepatitis B surface antigen (HBsAg) [118] which is considered to be a biomarker of liver cancer. This new immunosensor, which is based on the formation of trimetallic NiAuPt nanoparticles (NiAuPt-NGs) on reduced graphene oxide nanolayers, has intrinsic label-free properties since it can take amperometry-based measurements. In the study, NiAuPt-NG nanocomposite, which was previously treated with antibodies, was directly immobilized on the GO surface. Then, the electrochemical responses were evaluated before and after binding between antigen and antibody. It was determined that the trimetallic NiAuPt-NG nanocomposite material used in this design showed more electrocatalytic activity against hydrogen peroxide (H_2_O_2_) reduction than monometallic Pt-NG or bimetallic NiPt-NG nanocomposites. In addition, it has been determined that NiAuPt-NG is a good modification tool for antibodies and this nanocomposite amplifies the response signal. Under optimal conditions, the developed electrochemical immunosensor provides detection in the HBsAg assay in the linear range of 0.001 to 80 ng/mL and the detection limit of 0.31 pg/mL.

##### Label-Free but Redox Probe [Fe(CN)_6_]^3−/4−^-Based Voltammetric/Impedimetric Biosensors

**Figure 17:**
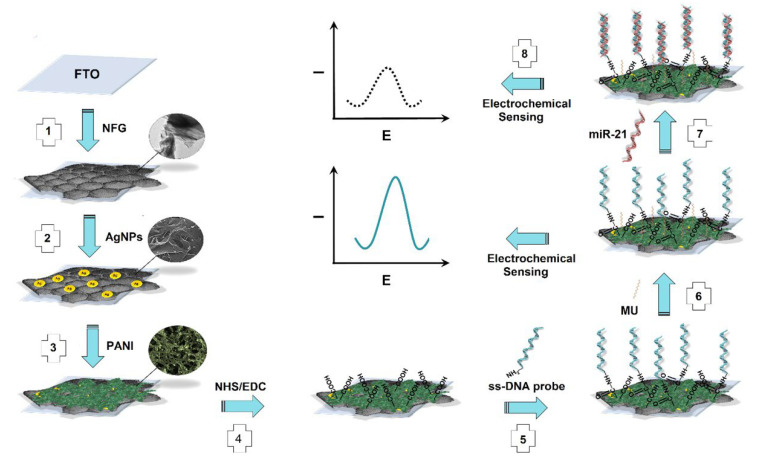
The schematic illustration of sensor fabrication; (1) Preparation of N-doped functionalized graphene (NFG)-modified fluorine-doped tin oxide (FTO) electrode. (2) Modification of silver nanoparticles and (3) PANI by electrodeposition. (4) Surface functionalization by using N-hydroxysuccinimide (NHS)/1-Ethyl-3-(3-dimethylaminopropyl) carbodiimide hydrochloride (EDC). (5) Immobilization of probe DNA. (6) Surface blocking process with 11-mercapto-1-undecanol (MU). (7) Hybridization with miRNA-21 target. (8) Measurement with differential pulse voltammetry (DPV) technique. Reprinted with permission Ref. [119]. Copyright 2018, Elsevier.

A sensitive and label-free nano-genosensor was reported by Salahandish et al. (Figure 17) for the detection of miRNA-21 sequence, a breast cancer biomarker, by using a specific sensing platform which contains nitrogen-doped functionalized graphene (NFG), silver nanoparticles (AgNPs), and polyaniline (PANI) structures [119]. Measurements performed both in the modification of the relevant layers to the electrode surface and before and after the hybridization between the amino group-labeled probe DNA and the target miRNA-21 were taken in potassium ferri/ferrocyanide redox probe solution and with EIS/DPV techniques. A sharp decrease in DPV voltammetric response of ferricyanide signal after interaction between probe and target sequences proved the hybridization event. The three-layer nanocomposite structure used in the design of the electrode surface, after the optimum conditions were carefully found, enabled the biosensor to reach a very low detection limit of 0.2 fM. Thanks to the related structure, it was possible to work in a very wide dynamic detection range of 10 fM–10 μM in the determination performed with the binding of more capture DNA molecules to the surface and increased electron transfer. This situation suggests that the developed graphene-containing nanobiosensor system may be very suitable for the early diagnosis of breast cancer.

The electrochemical impedance spectroscopy (EIS) technique is one of the most preferred platforms for aptamer–antigen interaction-based analysis because of its high selectivity, sensitivity, and label-free method. In this context, an aptasensor study was conducted by Ingebrandt’s group for the determination of prostate specific antigen (PSA) without using any redox markers and using interdigiated electrode (IDEs) coated with reduced graphene oxide (rGO) thin films as a transducer [120]. In IDE chips prepared by the photolithography technique, there are 16 individual drain electrodes (16 channels) sharing a common source electrode, and the resistance and capacitance of each electrode were designed to be compared. Graphene oxide was modified on these electrode surfaces by a spin coating technique (about 8 nm thickness). For biomolecule immobilization to the IDE surface, first the rGO layer was activated to carry -COOH group, and then the amino-linked PSA aptamer was covalently attached to the surface. Then, a buffer solution containing various concentrations of PSA antigen was sent to the electrode surfaces in the fluidic system for analysis, and changes in the resistance of rGO thin films were detected as a result of aptamer-antigen binding. The detection limit was calculated to be lower than 33pM for this device with a large linear detection range of 0.033–330 nM. It was predicted by the authors that this sensor is promising for the detection of PSA in real samples as well as the simultaneous detection of different cancer biomarkers.

An interesting label-free aptamer-based DNA biosensor strategy has been successfully applied by Aayanifard and colleagues for the sensing of prostate-specific antigen (PSA) for early detection of prostate cancer [121]. In the system in which the glassy carbon electrode is used, Aptamer/Au/GO modification was made on the biosensor surface and thus the biosensor gained the ability to capture PSA antigen. After aptamer antigen binding, measurements were performed using ferrocyanide solution using cyclic voltammetry, square wave voltammetry, and electrochemical impedance spectroscopy methods. In the study, TiO_2_/carbon quantum dots (CQDs) were subsequently added to the ferrocyanide solution with different pH values of 5.4 and 8, which was used for the first time in the measurement for the discrimination of cancerous and benign antigens. In line with this strategy, it was attempted to determine the total and free PSA separately in the analysis. The detection limit of the designed aptasensor under optimum conditions was 0.007 ng/mL and its dynamic concentration range was 0.5–7 ng/mL.

**Figure 18:**
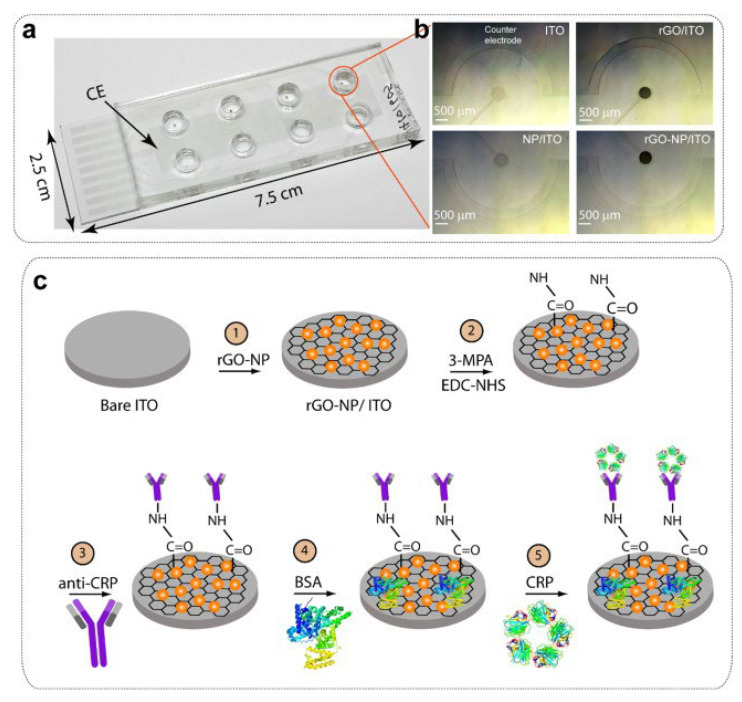
(**a**) Fabrication of the of the eight-channel microdisk array biosensor. It consists of rGO-AuNP/ITO-based circular working electrodes and only one counter electrode. (**b**) Optical images of the bare and modified electrodes along with a large ITO counter electrode. (**c**) Nanomaterial modifications of the ITO (rGO-NP functionalization) electrode and the preparation of the biosensing surface (EDC-NHS-based CRP antibodies binding on MPA-coated rGO-NP. Reprinted with permission from Ref. [122]. Copyright 2016, Elsevier.

An indium tin oxide (ITO) electrode-based innovative impedimetric immunosensor modified with reduced graphene oxide-gold nanoparticle (rGO-AuNP) hybrid structure was presented by Yagati et al. [122] for the detection of C-Reactive Protein (CRP), an important biomarker especially used in the diagnosis of cardiovascular diseases, infectious diseases, cancer diseases, etc., and also in monitoring the response to treatment. In the study, graphene-Au nanocomposites were modified by electrodeposition on the chip surface of the eight-channel microdisk array (MDEA) containing eight circular working electrodes produced by the photolithography technique. Then, by creating a self-assembled monolayer on the surface by using 3-mercaptopropionic acid, antibody immobilization to the sensor was performed by carbodiimide chemistry (Figure 18). After the application of the surface-blocking protocol, antigen analysis was monitored directly with an electrochemical analyzer by using CV and EIS techniques. The analysis was repeated in real serum samples diluted at a ratio of 1:200 and direct CRP determination could be performed label-free. It was determined that the surface-modification process containing rGO-NP in the array system developed in the study increased the electron transfer. In addition, it has been evaluated that it is a very suitable nanohybrid for high-rate biomolecule immobilization to the biosensor surface. The linear detection range of the developed sensor is 1 ng/mL and 1000 ng/mL, and the detection limit is calculated as 0.06 and 0.08 ng/mL for buffer and serum sample, respectively. Based on these findings, it was emphasized that the design with a stable surface has features that can be used in point-of-care applications for multiplex assays.

Kumar et al. [123] reported a label-free biodetection platform for the detection of CYFRA-21-1, an oral cancer biomarker, based on the sensing surface containing zirconia nanoparticles (mean particle size 13 nm) attached to reduced graphene oxide (ZrO_2_–rGO). In the biosensor where ITO electrode is preferred, functionalization was achieved with APTES molecule (3-aminopropyl triethoxy silane) after surface enrichment with zirconia (ZrO_2_) and rGO. Then, after the antibody immobilization, surface blocking, and antibody–antigen interaction processes were performed on the sensor surface, respectively, measurements were taken using the DPV method in potassium ferri/ferro cyanide solution. The interference analyses were also performed in real samples using salivary fluid obtained from six oral cancer patients. In addition, the enzyme linked immunosorbent assay (ELISA) method was used as the gold standard and comparisons were made with the newly developed method. This immunosensor, which can be used non-invasively, has a detection limit of 0.122 ng/mL and a sensitivity of 0.756 microampere mL/ng. It has been reported that the linear range in the determination is 2–22 ng/mL, the response time is 16 min, and the surface stability time is 8 weeks.

**Figure 19:**
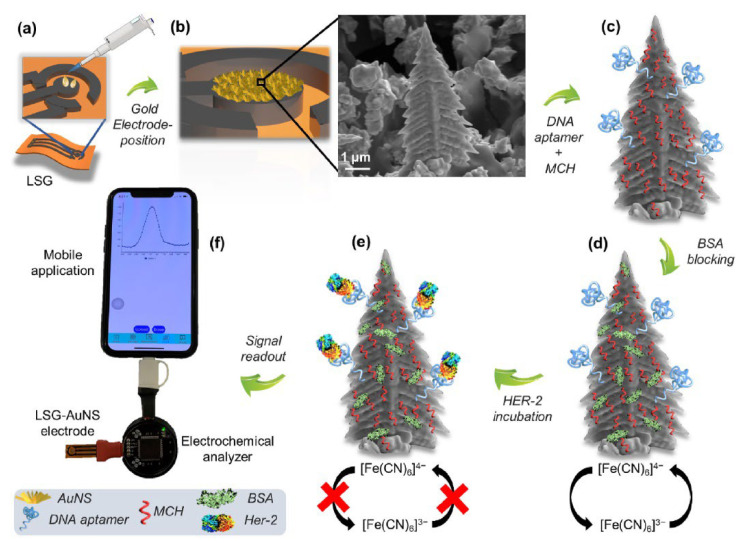
Schematic presentation of laser-scribed graphene-based (LSG) biosensor. (**a**,**b**) The modification of LSG electrode with AuNS by electrodeposition (Inset: formation of spiky and Christmas tree-like gold nanostructures). (**c**) Modification of biomolecule (DNA aptamer) onto the electrode by self-assembly using mercaptohexanol (MCH). (**d**) Surface blocking with BSA. (**e**) Incubation with the HER-2 antigen. (**f**) Measurement of the electrochemical signal caused by [Fe(CN)_6_]^3^/[Fe(CN)_6_]^4−^ redox probe. Reprinted with permission from Ref. [124]. Copyright 2021, Elsevier.

As another interesting example of graphene-containing biosensors in cancer research, Rauf et al. reported [124] a laser-scribed graphene (LSG)-based new generation electrode for the detection of human epidermal growth factor receptor 2 (HER-2). In the study, graphene-based working, reference, and counter electrode structures were prepared by printing on a flexible polyimide sheet with laser printing technology (LSG). Then, gold nanostructures, AuNS (having a unique morphology of spiky and Christmas tree-like 3D shapes) were modified by electrodeposition on the surface of the working electrode (Figure 19). This particular sensing surface was then modified by the thiol-labeled HER-2 aptamer and blocked with BSA to close the surface to undesired biomolecule binding. Then, at the analysis stage, HER-2 protein was interacted with LSG-AuNS/aptamer electrodes for incubation as the analyte. Altered electrochemical responses before and after HER-2 binding were investigated in conventional [Fe(CN)_6_]^3−^/[Fe(CN)_6_]^4−^ containing redox probe solution using CV and SWV techniques. As expected, a decrease in electrochemical response was obtained after the target analyte was bound. The detection limit of the developed LSG-AuNS-based aptasensor was found to be 0.008 ng/mL. Thanks to the 3D gold nanostructures deposited on LSG, high electron transfer rate, low detection limit, and reproducible results were achieved. Another outstanding aspect of this study is the development of an aptasensor that can accurately and sensitively analyze the amount of HER-2 added to undiluted human serum. In addition, a special software has been developed to use the presented system as a POC biosensor device, and the conversion from a laboratory aptasensor to a hand-held aptasensor containing a mobile phone has been achieved. However, with the handheld device, analysis could be performed with a higher detection limit (100 ng/mL) than the aptasensor, which was developed in this study for use only in the laboratory environment.

Analysis of cancer cells or their markers using modified graphene oxide with different metallic structures such as tungsten oxide-graphene composite [125] and reduced graphene oxide–tetra ethylene pentamine and trimetallic AuPdPt nanoparticles combination [126] has also been preferred in biosensor methodologies.

**Figure 20:**
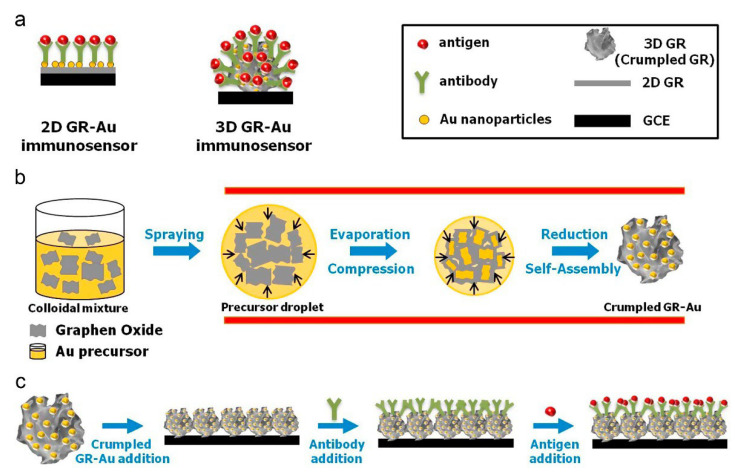
(**a**) Schematic representation of electrodes containing 2D GR–Au and 3D GR–Au, (**b**) preparation of wrinkled GR–Au composites using aerosol spray pyrolysis method, and (**c**) immunosensor surface modification with related composite materials and biomolecules. Reprinted with permission from Ref. [127]. Copyright 2015, Elsevier.

Biosensor designs with 3D materials have also attracted attention in recent years. To give an example of a prominent work, Jang et al. [127] developed a three-dimensional (3D) electrochemical immunosensor design for prostate-specific antigen (PSA) analysis and used conductive graphene (GR)-based gold (Au) composite-modified electrode in their sensor. In the study, the effects on the determination were investigated by using both 2D graphene and 3D graphene (crumpled graphene ball). Electrochemical impedance spectrometry was used for the surface characterization of the developed 2D and 3D graphene electrode structures (Figure 20). Analysis based on the measurements taken by cyclic voltammetry technique in ferri/ferrocyanide solution was carried out by the interaction of the PSA antigen with the anti-PSA antibody immobilized on the glacial carbon electrode coated with this composite structure with an improved surface area and increased conductivity. The linear range of this 3D crumpled GR ball immunosensor is 0–10 ng/mL and the detection limit is calculated as 0.59 ng/mL.

For more information on electrochemical biosensors developed for cancer diagnosis, both containing types of graphene and with other nanomaterial design contents, some valuable review studies reported recently are available in the literature [18,75,77,90,128,129,130,131,132].

Graphene oxide-based nanomaterials on immunosensors or biosensors offer a range of possibilities in regard to cancer diagnosis. Two tables containing some of the other recently reported studies and the basic biosensor design elements related to these studies is presented below (Table 1 and Table 2).

**Table 1:** Label/redox mediator/indicator-based graphene oxide containing electrochemical biosensors for cancer diagnostics.

Sensor Surface Modification	Capture Biomolecule for Sensing	Detected Cancer Biomarker	Electrochemical Technique Involved in the Marker Determination	Linear Range and LOD	Reference
Glassy carbon electrode (GCE) functionalized with porous GO/Au composites and porous PtFe alloy	MUC1- Aptamer	MCF-7 cells (breast cancer)	EIS, CV, DPV	100–5.0 × 10^7^ cells/mL; 38 cells/mL	[133]
Au/Ti coated Si/SiO_2_ substrate modified with trimetallic Pd@Au@Pt nanocomposites platform on -COOH terminated reduced graphene oxide (COOH-rGO)	Antibody	CEA (different cancers) PSA (Prostate cancer)	CV, DPV	12 pg/mL–85 ng/mL; 8 pg/mL (CEA) 3 pg/mL–60 ng/mL; 2 pg/mL (PSA)	[134]
Screen-printed carbon electrodes (SPCEs) functionalized with a water-soluble reduced graphene oxide− carboxymethylcellulose (rGO-CMC) hybrid nanomaterial	Hairpin capture DNA probes	p53 tumor suppressor (TP53) gene (different cancers)	Amperometry	0.01−0.1 μM; 3.4 and 2.9 nM for two capture probes	[135]
Screen-printed carbon electrodes (SPCEs) functionalized with superparamag- netic graphene-loaded iron oxide nanoparticles (GO-NPFe_2_O_3_).	None (based on graphene-RNA affinity interaction)	FGFR2:FAM76A fusion gene (ovarian cancer)	Coulometry	Unspecified; 1 fM	[136]
Electrophoretic deposition (EPD) of reduced graphene oxide (rGO) onto a gold electrode and post-functionalization of rGO with folic acid (Au/rGO-FA)	none	Folic acid protein (human epithelial-derived cancers)	DPV	1–200 pM ; 1 pM	[137]
Screen-printed carbon electrodes (SPCEs) functionalized with graphene oxide-loaded iron oxide (GO/IO hybrid material)	None (via RNA- graphene oxide (GO) affinity interaction)	miR-21 (ovarian cancer)	Coulometry, Amperometry, CV	1.0 fM–1.0 nM; 1 fM	[138]
Glassy carbon electrode (GCE) functionalized with IL-rGO–Au	Antibody	CEA (different cancers) AFP (Hepatocellular cancer)	SWV	0.01–100 ng/mL; 0.003 and 0.002 ng/mL	[139]
Screen-printed carbon electrodes (SPCEs) functionalized with carboxylic group (-COOH) rich graphene oxide (GO)	Antibody	MUC1 (variety of cancers)	DPV	0.1–2 U/mL; 0.04 U/mL	[140]
Au electrode functionalized with graphene oxide/ polyaniline nanostructures (GO-PANI) and Au NPs.	Antibody	Nuclear matrix protein 22 (NMP22), CEA (different cancers)	SWV	0.1 pg/mL–0.3 ng/mL; 25 and 30 fg/mL	[141]
Glassy carbon electrode functionalized with sulfur-doped reduced graphene oxide (SrGO)	None (direct 8-hydroxy-2′- deoxyguanosine (8-OHdG) signal measurement)	8-OHdG molecule (cancer)	CV, EIS, DPV	2 nM–20 μM; 1 nM	[142]
Glassy carbon electrode functionalized with magnesium oxide (MgO) nanoflower and gold nanoparticles (AuNPs). (GO is in the label, not in the surface modification)	Thiolated capture DNA probe	miRNA-21 (different cancers)	CV, EIS, DPV	0.1–100 fM; 50 aM	[143]
Glassy carbon electrode functionalized with reduced graphene oxide-chitosan (rGO-Chit) film	Aptamer	HER2 (breast cancer)	DPV	0.5–2 ng/mL and 2–75 ng/mL (two linear concentration ranges); 0.21 ng/mL	[144]
Glassy carbon electrode functionalized with Au- poly(methylene blue) (Au-PMB) and reduced graphene oxide-Au nanocomposites (Au-rGO)	Peptides (CEHSSKLQLAK-NH_2_)	PSA (Prostate cancer)	SWV	1.0 fg/mL–100 ng/mL; 0.11 fg/mL	[145]
Gold electrode functionalized with MPA and capture antibody (GO is in the label, not in the surface modification)	Antibody	Du-145 cells (prostate metastatic cancer)	DPV	10^2^–10^6^ cells/mL; 20 cells/mL	[146]
Screen-printed carbon electrodes (SPCEs) functionalized with graphene oxide (GO) and ex-situ prepared silver nanoparticles (AgNPs)	Antibody	PSA (Prostate cancer)	CV, EIS, DPV	0.75–100 ng/mL; 0.27 ng/mL	[147]

**Table 2:** Label-free graphene oxide-based electrochemical biosensors for cancer diagnostics.

Sensor Surface Modification	Capture Biomolecule for Sensing	Detected Cancer Biomarker	Electrochemical Technique Involved in the Marker Determination	Linear Range and LOD	Reference
Paper-based electrode functionalized with silver nanoparticles-reduced graphene oxide nanocomposite (Ag/RGO) ink and cysteamine coped gold nanoparticles (CysA/Au NPs)	Antibody	CA15-3 protein (breast cancer)	Amperometry	15–125 U/mL; 15 U/mL (LLOQ)	[148]
ITO coated glass electrode functionalized with palladium nanoparticle decorated-reduced graphene oxide (Pd@rGO)	Antibody	PSA (Prostate cancer)	CV, EIS, Amperometry	0.01–12.5 ng/mL; 10 pg/mL	[149]
Glassy carbon electrode functionalized with palladium-reduced graphene oxide (Pd–rGO)	Antibody	AFP (Hepatocellular cancer)	Amperometry, DPV	0.01–12 ng/mL; 5 pg/mL	[31]
Glassy carbon electrode functionalized with nanocomposite of Au NPs decorated on aminated reduced graphene oxide (Au–NH_2_–rGO)	Antibody	PSA (Prostate cancer)	Amperometry, EIS	0.5 pg/mL–15 ng/mL; 0.17 pg/mL	[150]
Fluorine tin oxide (FTO) sheets coated with carboxylated graphene oxide followed by deposition of gold- platinum bimetallic nanoparticles	Capture DNA probe	miRNA-21 (breast cancer)	CV, EIS, DPV	1 fM–1 μM; 1 fM	[151]
Fluorine-doped tin oxide (FTO) electrodes functionalized with facet-controlled Au nanorods-functionalized reduced graphene oxide (Au NRs/rGO)	Antibody	PSA (Prostate cancer)	CV, EIS, DPV	0.1–150 ng/mL; 0.016 ng/mL	[152]
Pencil graphite electrode functionalized with graphene oxide (GO)	Capture DNA probe	miRNA-34a (different cancers)	CV, EIS	0–10 μg/mL; 1.84 μg/mL (261.7 nM)	[153]
Glassy carbon electrode functionalized with rhombic dodecahedral Cu_2_O nanocrystals– graphene oxide– gold nanoparticles (rCu_2_O-GO-AuNPs)	Antibody	CEA (different cancers)	CV, EIS	0.01–120 ng/mL; 0.004 ng mL	[154]
Glassy carbon electrode functionalized with Au loaded on thionine functionalized graphene oxide (Au@Th/GO)	Antibody	PSA (Prostate cancer)	Amperometry, CV, EIS	50 fg/mL–40 ng/mL; 16.6 fg/mL	[155]
Glassy carbon electrode functionalized with reduced graphene oxide /gold nanoparticles (GO/AuNPs) and antibody.	Antibody	PSA (Prostate cancer)	CV, EIS, SWV	Unspecified; 0.2 and 0.07 ng/mL for total and free PSA antigen	[156]
ITO coated glass electrode functionalized with cerium oxide nanocubes (ncCeO_2_)– reduced graphene oxide (RGO)-based nanocomposite.	Antibody	Cyfra-21-1 (oral cancer)	CV, EIS, DPV	0.625 pg/mL–15 ng/mL; 0.625 pg/mL	[157]
Platinum electrode functionalized with GO layers and AuNPs.	Antibody	PSA (Prostate cancer)	CV, DPV, EIS	0.001 fg/mL–0.02 μg/mL; 0.24 fg/mL	[158]
Glassy carbon electrode functionalized with herceptin-conjugated graphene.	Antibody	HER2 (breast cancer)	EIS, CV, DPV	1–80 cells ; Unspecified	[159]
Graphite electrode functionalized with reduced graphene oxide nano-sheets (rGONs) and rhodium nanoparticles (Rh-NPs).	Aptamer	HER2-ECD (breast cancer)	CV, DPV, EIS	10.0–500 ng/mL; 0.667 ng/mL	[160]
Au film electrode functionalized with Fe_3_O_4_@graphene oxide(GO)@molecularly imprinted polymer (MIP) nanoparticles.	Special sensor surface structure originating from MIP	IL-8 (oral cancer)	CV	0.1–10 pM ; 0.04 pM	[161]
ITO coated glass electrode functionalized with zinc oxide–reduced graphene oxide (ZnO–rGO) nanocomposite.	Antibody	IL-8 (oral cancer)	CV, DPV	100 fg/mL– 5 ng/mL; 51.53 ± 0.43 pg/mL	[162]
Pencil graphite electrodes modified with carbon black, multi-walled carbon nanotubes, and graphene oxide nanomaterials.	Capture DNA probe	microRNA-125a (different cancers)	CV, EIS	0.008 and 15 μg/mL; 10 pM	[163]
FET chip functionali-zed with gold nano- particles (AuNPs)- decorated reduced graphene oxide.	Peptide nucleic acid (PNA) probe	miRNA (type not specified)	FET	Unspecified ; 10 fM	[164]
FET channel functionalized with tetra(4-aminophenyl) porphyrin mediated reduced graphene oxide.	Aptamer	CTCs (different cancers)	FET	10–10^6^ cells/mL ; Unspecified	[165]
Graphene FET chip functionalized with 1-pyrenebutyric acid N-hydroxysuccinimide ester (PBASE).	Antibody	AFP (hepatocellular cancer)	FET	Unspecified; 0.1 ng/mL in PBS, 12.9 ng/mL in plasma	[166]
Pentacene-based FET with a graphene oxide support system (GOSS), composed of functionalized graphene oxide (GO) ink.	Single stranded DNA/ Antibody	Target DNA/ CTCs (basically HER2, breast cancer)	FET	Unspecified; 0.1 pmoles for DNA and 100 cancer cells/mL	[167]
FET functionalized with carboxylated multiwalled carbon nanotubes (MWCNTs)/ reduced graphene oxide.	Aptamer	CA 125 (ovarian cancer)	FET	1.0 × 10^−9^–1.0 U/mL ; 5.0 × 10^−10^ U/mL	[168]
FET functionalized with Polymethyl methacrylate(PMMA) and graphene films	Antibody	CEA (different cancers)	FET	Unspecified; less than 100 pg/mL	[169]

## 2. Challenges and Future Outlook

In this review, we tried to present prominent graphene oxide-based electrochemical studies that have been reported in recent years and have a suitable infrastructure for clinical use for cancer diagnosis. Although significant progress has been made in the synthesis of graphene and its derivatives and their use in drug carriers/therapeutics in vitro, more progress is needed in the field of in vivo analysis. On the other hand, if an evaluation is made in terms of electrochemical studies, first of all, it can be said that while promising studies continue, there are also discussions on whether graphene and its derivatives increase the electroactivity/conductivity sufficiently. For this purpose, many elements, including N, S, P, B, etc., are integrated into graphene structures by doping [170]. We believe that this technology will come to the fore in order to increase the performance of new biosensors yet to be developed, and with the increase of electrocatalytic activity, successful designs that can be used in clinical and POC applications will be revealed.

As is known, zero-dimensional graphene quantum dots GQDs, a member of the latest-generation graphene family, have also attracted great interest from researchers and industries in recent years [171]. In line with the developments in this field, it is thought that graphene oxide and reduced graphene oxide nanomaterials in biosensors will replace them, and it is predicted that the potential of clinical validity in cancer diagnosis will be great, based on the wide linear detection ranges and low detection limits of GQDs-based biosensors reported so far.

Apart from all these, comprehensive studies including extensive efforts in the field of theranostics, which combine diagnosis and treatment in a single system/platform in the medical field, are also being carried out [172,173]. Having expectations and goals for the development of such high-quality device systems should lead researchers to the design of electrochemical biosensors that can be converted into products.

It is necessary to develop at least stable surface sensors and diagnostic kit for “open–use–dispose”-type electrochemical sensors [174]. Much more intensive efforts are needed to develop EC biosensors that will provide fast and accurate analysis with a single easy measurement, similar to optical sensors and blood glucose or pregnancy test systems, in accordance with the requirements of the current era. In this context, “rapid diagnostic kits” based on antigen–antibody binding developed during the COVID-19 pandemic have demonstrated how important it is to develop diagnostic kits today. As it was preferred in the development of these tests, most of the biosensing systems produced until today are based on “immunosensors”. We believe that this design technology will also be preferred in new-generation wearable biosensor systems because the size of the analytes determined indicates that, for faster and more sensitive determination, immunosensor technology is more suitable than DNA sensors or aptasensors.

Finally, we would like to point out that we think that, among the electrochemical biosensors examined in this review, paper-based sensors or systems that can measure with an application able to be downloaded to a mobile phone are more suitable for clinical practice. However, electrochemical biosensors (i.e., GFETs, other label-free systems) are also very promising for wearable health monitoring, thanks to their important advantages such as selectivity, sensitivity, rapidity, portability [175]. It is clear that wearable biosensors and self-powered devices will be the future of design in the field of disease diagnosis and monitoring. Therefore, it is essential to develop easily manufacturable electrochemical biosensors that provide accurate analysis with a small amount of material rather than complex designs where multiple modifications are made on the electrode surface. Of course, in such wearable biosensor designs, the lifetime parameter should be carefully evaluated.

## Abbreviations

Gr or GR, Graphene; GO, Graphene oxide; rGO, Reduced graphene oxide; GQD, graphene quantum dots; CQD, carbon quantum dots; CVD, chemical vapor deposition; SNP, single nucleotide polymorphisms; TMB, (3,3,5,5-tetramethylbenzidine; NMR, Nuclear Magnetic Resonance; EDC, 1-(3-dimethylaminopropyl)-3-ethyl carbodiimide hydrochloride; NHS, N-Hydroxysuccinimide; Rs, electrode/electrolyte solution resistance; Zw, Warburg impedance; Cdl, double layer capacitance; Ret, electron transfer resistance; GCE, glassy carbon electrode; CV, cyclic voltammetry; EIS, electrochemical impedance spectroscopy; DPV, differential pulse voltammetry; miRNA, mikro RNA; CEA, carcinoembryonic antigen; AFP, alpha-fetoprotein; CGS, carboxyl graphene nanosheets; TB, toluidine blue, PB, Prussian blue, EpCAM, epithelial cell adhesion molecule; ITO, indium tin oxide; quantum dots (QDs); SWASV, square wave anodic stripping voltammetry; LOD, limit of detection; TiP, titanium phosphate nanospheres, Ru(NH_3_)_6_^3+^, ruthenium hexamine; SWV, square wave voltammetry; CTCs, circulating tumor cells; AuNPs, gold nanoparticles; μPAD, paper-based analytical device; HRP, horseradish peroxide; SiO_2_ or SiO_2_ NPs, silica nanoparticles; PH-AuPd NP, polyhedral AuPd alloy nanoparticles; PWE, paper working electrode; PSA, Prostate-specific antigen; PEDOT, poly (3,4-ethylenedioxythiophene); THI, thionine; MB, methylene blue, POC or POCT, point-of-care applications; MBs, magnetic beads; SPCE, screen-printed carbon electrode; FET, field effect transistor; GFET or Gr-FET, graphene field effective transistor; SA, streptavidin; LAPS, light addressable potentiometric sensor; APTES, 3-aminopropyl triethoxy silane; graphene fiber (GF) NWs, nanowires; NGs, graphene nanosheets; IDEs, interdigiated electrode; MPA, mercaptopropionic acid, LOQ, limit of quantitation; lower limit of quantitation (LLOQ); IL-8, interleukin 8.
